# Tracking the evolutionary history of *Cortinarius *species in section *Calochroi*, with transoceanic disjunct distributions

**DOI:** 10.1186/1471-2148-11-213

**Published:** 2011-07-19

**Authors:** Sigisfredo Garnica, Philipp Spahn, Bernhard Oertel, Joseph Ammirati, Franz Oberwinkler

**Affiliations:** 1 Organismic Botany, Institute of Evolution and Ecology, University of Tübingen, Auf der Morgenstelle 1, D-72076 Tübingen, Germany; 2 Division of Animal Genetics, University of Tübingen, Auf der Morgenstelle 28, D-72076 Tübingen, Germany; 3 INRES Gartenbauwissenschaft, University of Bonn, Auf dem Hügel 6, D-53121 Bonn, Germany; 4 Department of Biology, Box 351330, University of Washington, Seattle, Washington 98195, USA

## Abstract

**Background:**

*Cortinarius *species in section *Calochroi *display local, clinal and circumboreal patterns of distribution across the Northern Hemisphere where these ectomycorrhizal fungi occur with host trees throughout their geographical range within a continent, or have disjunct intercontinental distributions, the origins of which are not understood. We inferred evolutionary histories of four species, 1) *C*. *arcuatorum*, 2) *C. aureofulvus*, 3) *C*. *elegantior *and 4) *C. napus*, from populations distributed throughout the Old World, and portions of the New World (Central- and North America) based on genetic variation of 154 haplotype internal transcribed spacer (ITS) sequences from 83 population samples. By describing the population structure of these species across their geographical distribution, we attempt to identify their historical migration and patterns of diversification.

**Results:**

Models of population structure from nested clade, demographic and coalescent-based analyses revealed genetically differentiated and geographically structured haplotypes in *C*. *arcuatorum *and *C*. *elegantior*, while *C*. *aureofulvus *showed considerably less population structure and *C. napus *lacked sufficient genetic differentiation to resolve any population structure. Disjunct populations within *C*. *arcuatorum, C. aureofulvus *and *C*. *elegantior *show little or no morphological differentiation, whereas in *C. napus *there is a high level of homoplasy and phenotypic plasticity for veil and lamellae colour. The ITS sequences of the type specimens of *C. albobrunnoides *and *C. albobrunnoides *var. *violaceovelatus *were identical to one another and are treated as one species with a wider range of geographic distribution under *C. napus*.

**Conclusions:**

Our results indicate that each of the *Calochroi *species has undergone a relatively independent evolutionary history, hypothesised as follows: 1) a widely distributed ancestral population of *C*. *arcuatorum *diverged into distinctive sympatric populations in the New World; 2) two divergent lineages in *C*. *elegantior *gave rise to the New World and Old World haplotypes, respectively; and 3) the low levels of genetic divergence within *C*. *aureofulvus *and *C*. *napus *may be the result of more recent demographic population expansions. The scenario of migration via the Bering Land Bridge provides the most probable explanation for contemporaneous disjunct geographic distributions of these species, but it does not offer an explanation for the low degree of genetic divergence between populations of *C. aureofulvus *and *C. napus*. Our findings are mostly consistent with the designation of New World allopatric populations as separate species from the European counterpart species *C. arcuatorum *and *C. elegantior*. We propose the synonymy of *C. albobrunnoides*, *C. albobrunnoides *var. *violaceovelatus *and *C. subpurpureophyllus *var. *sulphureovelatus *with *C. napus*. The results also reinforce previous observations that linked *C. arcuatorum *and *C. aureofulvus *displaying distributions in parts of North America and Europe. Interpretations of the population structure of these fungi suggest that host tree history has heavily influenced their modern distributions; however, the complex issues related to co-migration of these fungi with their tree hosts remain unclear at this time.

## Background

Several investigators [1-13] have explored intercontinental patterns of ectomycorrhizal fungus species distributions in a phylogenetic context, but there is little information on the origin and patterns of speciation in these fungi. Mushrooms in the genus *Cortinarius*, section *Calochroi *(calochroid clade), with over 100 species, represent one of the most conspicuous ectomycorrhizal members of contemporary Northern Hemisphere forest ecosystems [14]. Previous studies of species in *Calochroi *show various patterns of geographic distributions, including local (*C. cisticola*), clinal (*C. arcuatorum*) or circumboreal (*C. aureofulvus*, *C. aureopulverulentus*, *C. cupreorufus*, *C. elegantior*) distributions. In most instances fungus species distributions correspond to that of their host trees within continents and often disjunct distributions across the Northern Hemisphere show a similar pattern [15-18]. Overall, the patterns of species distribution in the *Calochroi *roughly matches the range of genera of the host tree families Fagaceae (*Castanea, Castanopsis, Chrysolepis, Fagus*, *Notholithocarpus*, *Quercu*s) and Pinaceae (*Abies*, *Larix*, *Picea*, *Pinus, Pseudotsuga*, *Tsuga*) [17-21], however, a few *Calochroi *species form ectotrophic associations with members of the Betulaceae (*Alnus*, *Corylus*, *Carpinus*) [22,23], Cistaceae (*Cistus*, *Helianthenum*) [24,25] and Malvaceae (*Tilia*) [24,25]. Host specificity of *Calochroi *species may be restricted to a single host species, with host-switching events being less common and often restricted to closely related plant species [17,18,20]. Interestingly, allopatric populations/species on an intercontinental scale display relatively high host fidelities at the host genus level, suggesting potential co-evolutionary/co-migratory trends [14].

Species with a broad distribution provide excellent models for examining their present patterns of spatial variation, especially where there is an obligate association as with an ectotrophic fungus. An evaluation of contemporary geographic patterns of these species can provide a better understanding of speciation and habitat requirement as well as determine host preference and host-switching events that have occurred over time. To date a major difficulty in many mushroom genera, including species of *Cortinarius*, section *Calochroi *is the difficulty of identifying species [14,26], which historically has been a major impediment to understanding their patterns of geographical distribution, ecology, diversity and evolutionary relationships. There are a limited number of unique morphological features [14] that can be used to construct robust evolutionary hypotheses within and between closely related species in section *Calochroi*. Based on similarities in basidiomata morphology and colour some widespread species with largely allopatric distributions in the Old World (Europe) and the New World (North America) (e.g. *C*. *arcuatorum*, *C*. *aureofulvus*, *C*. *aureopulverulentus*, *C*. *cupreorufus *(as *C. orichalceus*), and *C. olivellus*) were treated as conspecific [17,18,20]. On the other hand, the discovery of certain phenotypic differences between European populations and their North American counterparts has led to the recognition of infraspecific taxa, for example *C. elegantior *var. *americanus *[17]. Similarly, detailed observations of morphological variation between European collections of *C. elegantior *have resulted in a number of forms, subspecies and varieties [19]. In our recent phylogenetic study [14], we confirmed close phylogenetic affinities between some disjunct populations/species, but we also found that several European species and their disjunct North American counterparts showed significant divergence from one another.

Recently the use of DNA sequence data have provided an independent tool for delineating species boundaries, and for revealing patterns of evolution history, identifying convergent morphological features and assessing/estimating species diversity. An evaluation of ITS rDNA sequences published by us [14] revealed distinct intra- and/or inter-continental phylogeographic structuring within some species, sometimes accompanied by relatively little morphological divergence. In contrast, we also found relatively low levels of genetic divergence within and between species with allopatric distributions in North America and Europe that display substantial colour variation in major components of the basidiomata. While, considerable progress has been made in reconstructing interspecific relationships within the section *Calochroi *through analyses of morphological features and DNA sequences [14,26], however, further studies are needed to examine how sequence divergence relates to the separation or divergence of species over time at continental and inter-continental levels.

Differences in genetic structure among extant species can be used to infer their evolutionary histories. As previously stated, rapidly evolving ITS regions of rDNA from disjunct species of section *Calochroi *found in regions of Europe and North America show different levels of sequence divergence. However, to date there has not been sufficient data to draw consistent conclusions concerning the genetic variation and divergence of these species. For example, maximum likelihood (ML) analyses [14] suggest a complex evolutionary history within *C*. *arcuatorum*, including divergence into four distinctive monophyletic groups with similar morphological features. In contrast, a close phylogenetic relationship was found between species considered to be morphologically separate from one another; European *C. napus *and North American *C. albobrunnoides *(vars. *albobrunnoides *and *violaceovelatus *and *C. subpurpureophyllus *var. *sulphureovelatus*). To gain a more complete understanding of the evolutionary histories of *Calochroi *species (including phylogeographic structure, character variation, and gene flow), we selected four species for study: 1) *C*. *arcuatorum*, 2) *C*. *aureofulvus*, 3) *C*. *elegantior *and 4) *C*. *napus*. Our species concept is anchored in the morphology (macroscopic, chemical, microscopic characteristics) of the basidiomata or mushrooms, which is the structure associated with sexual reproduction of basidiospores. Morphological and molecular evidences show that the four species treated here are each clearly distinct from other known *Cortinarius *species. The four selected species are somewhat rare to uncommon (especially *C. aureofulvus *and *C. napus*) across their known geographical range. They are mainly restricted to more calcareous soils, exhibit intercontinental distributions within boreo-nemoral areas, or in the case of *C. arcuatorum *extend into in the meridional zones [14]. Across their known distributions, *C. arcuatorum *is associated with oak (*Quercus*), beech (*Fagus*), hornbeam (*Carpinus*) and tan oak (*Notholithocarpus*) species, and with spruce (*Picea*) and Douglas fir (*Pseudotsuga menziesii*) in Wyoming. The tree hosts grow from lowland regions to high elevations, up to 3000 m in Costa Rica, and up to 2200 m in Wyoming. Three species, *C. aureofulvus*, *C*. *elegantior *and *C. napus *are associated with conifers including spruce (*Picea*), fir (*Abies*), pine (*Pinus*), Douglas fir (*Pseudotsuga menziesii*) and hemlock (*Tsuga*) species in subalpine regions or, less commonly, in mid- to low elevation forests (western *elegantior *clade and *C. subpurpureophyllus *var. *sulphureovelatus *(= *C. napus*)). Although these host tree genera also occur in Asia, so far there is no survey of these *Cortinarius *species for this region for inclusion in our study.

**Figure 1:**
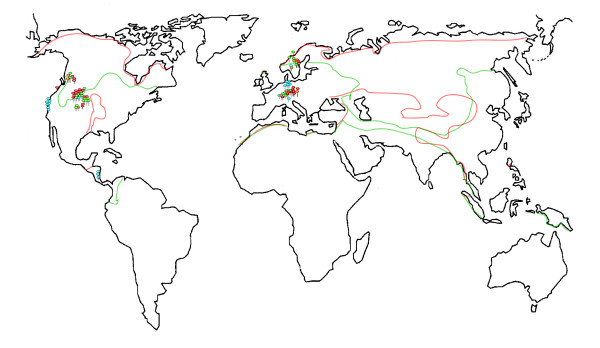
**A map showing the geographic location of the sampling sites of calochroid population samples across Europe, North America and Central America**. Population samples of *C. arcuatorum *are represented in blue, *C. aureofulvus *in orange, *C. elegantior *in red and *C. napus *in green. Collection numbers correspond to collections in Additional File 4. Distribution of host plant families: green dashed lines indicate distribution of Fagaceae and red dashed lines indicate distribution of Pinaceae, respectively.

Here to infer historical events associated with speciation, we collected population samples within four species over geographical distributions spanning Europe, North America and Central America (Figure 1), used nested clade, demographic and coalescent-based approaches to analyse the fast-evolving ITS region of the nuclear ribosomal DNA. Coalescent-based analyses incorporate random fluctuations in mutation, genetic drift and sampling errors into the calculation of demographic parameters, whereas these variations are underestimated by phylogenetic approaches [27]. We used these analyses to address the following specific questions. What are the mechanisms responsible for the contemporary patterns of geographic distribution within the four species, including centres of origin and potential migration routes? And, are species with allopatric distributions sufficiently distinct from one another within and between continents to merit a specific designation? To address these questions, we compared DNA sequence variation of species at continental and intercontinental scales. We also evaluated the phenotypical and ecological features of the *Calochroi *species. Special emphasis was placed on detecting highly labile traits and their patterns of geographic variation among population samples represented by individual collection containing one to several basidiomata from one group or cluster found growing in a relatively small patch (genet).

Our findings show that the comparison of population samples using phenotypical, ecological and genetic characteristics help to clarify questions concerning the historical events involved in the modern distributions of *Calochroi *species as well as aspects related to host preferences and host-switching, and the use of a combined phylogenetic, morphological and ecological species definition. We also discuss the utility and limitations of ITS sequences and phenotypic characteristics as identification tools for *Cortinarius *species with wide geographical distributions.

## Results

### DNA amplification, phylogenetic placement and relationship

**Figure 2:**
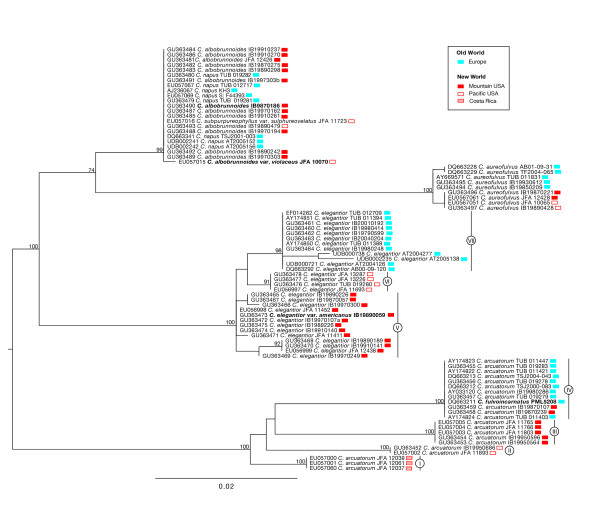
**Phylogenetic relationships of New World and Old World population samples of *Calochroi *species based on ML analysis of ITS rDNA sequences**. The represented tree was computed from 1000 runs and was mid-point rooted. Numbers above branches are bootstrap values (values < 70 not shown) from 1000 replicates. Assignations I to VII indicate major groups of *C. arcuatorum *and *C. elegantior *discussed in the text. Type specimens are printed in bold.

More than 100 fungus population samples, including our own collections and those from other herbaria, were analysed in this study (see the Methods section for more details). We were unable to amplify the ITS region for many collections because they were either poorly conserved or too old, and the sequence quality was too low, or we amplified contaminant fungi that probably colonised the dried basidiomata. Analysis of ITS sequences supported the phylogenetic placement of a total of 83 population samples identified as belonging to *C*. *arcuatorum*, *C*. *aureofulvus*, *C*. *elegantior *and *C. napus *lineages (data not shown). Each species was recovered as a monophyletic clade with high bootstrap support (Figure 2). The internal phylogeny of *C. arcuatorum *is composed of four major lineages: a clade including population samples from Costa Rica (I); a clade comprising population samples from Mendocino (Pacific USA) (II); a clade composed of samples from Del Norte Co (Pacific USA) (III); and a large clade containing mostly European and two sample populations from Wyoming (IV), respectively. Within *C. elegantior*, several population samples had unresolved positions (V), a portion of North American population samples are distributed into one well-supported subclade (VI) and European population samples formed a well-supported subclade (VII). The North American taxa *C. albobrunnoides*, *C. albobrunnoides *var. *violaceus*, and *C*. *subpurpureophyllus *var. *sulphureovelatus *and the European taxon *C. napus *formed a monophyletic group with not internal subclades. New World and Old World population samples of *C. aureofulvus *clustered together with no significant genetic differentiation between them.

### Patterns of nucleotide variation in the ITS region

**Figure 3:**
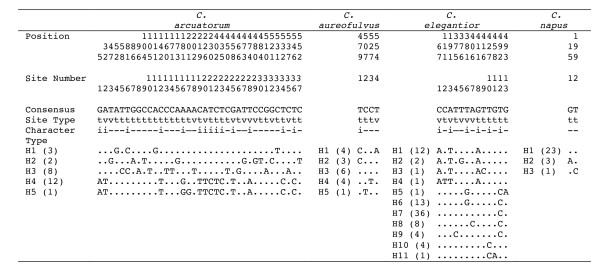
**Distribution of polymorphic sites after removing indels and recombination blocks in the ITS haplotypes of calochroid species**. t, transitions; v, transversions; i, phylogenetically informative sites; -, uninformative sites.

Sequence divergence of the complete ITS region between intercontinental disjunct populations ranged from identical to 4.63% in *C. arcuatorum*, from 0.16% to 0.35% in *C. aureofulvus*, from 0.68% to 1.46% in *C. elegantior *and from identical to 0.17% in *C. napus*. After removing indels and recombination blocks, twenty-four distinct ITS haplotypes were identified among the 83 population samples from the four species analysed: 5 haplotypes in *C*. *arcuatorum *and *C*. *aureofulvus*, 11 in *C*. *elegantior*, and 3 in *C. napus *(Figure 3). Nucleotide diversity (π) estimates for the whole population sample were 0.024 ± 1.9 × 10^-3 ^in *C*. *arcuatorum*, 0.002 ± 3.0 × 10^-4 ^in *C*. *aureofulvus*, 4.2 × 10^-3 ^± 4.2 × 10^-4 ^in *C*. *elegantior *and 5.0 × 10^-4 ^± 2.0 × 10^-4 ^in *C. napus *(Table 1). When species were partitioned into sampled areas, population samples showed similar or lower estimates of nucleotide diversity. The average per-nucleotide expected heterogeneity, θw, for the whole sample of each species was identical or higher than partitioned sampled areas.

**Table 1:** Population statistics, diversity estimates and neutrality tests based on ITS region of calochroid taxa studied

	Population statistics							Tests of neutrality			
**Species/** **population**	**n**	**s**	**h**	**hd**	**k**	**π** **(SD)**	**θw**	**Tajima's** **D**	**Fu and** Li's D* Statistic	**Fu and** Li's F* Statistic	**Fu's Fs** **Statistic**

*C. arcuatorum*											
Old World	11	1	2	0.182	0.181	3.3 × 10^-4^ (2.6 × 10^-4^)	6.1 × 10^-4^	-1.128 (NS)	-1.289 (NS)	-1.399 (NS)	-0.410 (NS)
New World	15	36	4	0.685	12.229	0.021	0.019	1.635 (NS)	1.502**	1.502 (NS)	9.684 (NS)
Mountain USA	2	0	1	0.000	0.000	0.000	0.000	ND	ND	ND	ND
Pacific USA	10	20	2	0.356	7.111	0.013 (5.6 × 10^-3^)	0.013	0.027 (NS)	1.567**	1.326 (NS)	9.432 (NS)
Costa Rica	3	0	1	0.000	0.000	0.000	0.000	ND	ND	ND	ND
All (l = 559)	26	37	5	0.698	13.741	0.024 (1.9 × 10^-3^)	0.017	1.569 (NS)	1.568**	1.843**	13.357 (NS)
*C*. *aureofulvus*											
Old World	7	1	2	0.571	0.571	0.001 (2.1 × 10^-4^)	7.3 × 10^-4^	1.341 (NS)	0.953 (NS)	1.101 (NS)	0.856 (NS)
New World	11	2	3	0.618	0.690	1.23 × 10^-3^ (2.9 × 10^-4^)	1.22 × 10^-3^	0.036 (NS)	-0.033 (NS)	-0.269 (NS)	-0.113 (NS)
Mountain USA	2	0	1	0.000	0.000	0.000	0.000	ND	ND	ND	ND
Pacific USA	9	2	3	0.667	0.777	0.001 (3.2 × 10^-4^)	0.001	0.195 (NS)	-0.221 (NS)	-0.135 (NS)	-0.108 (NS)
All (l = 560)	18	4	5	0.804	1.346	0.002 (3.0 × 10^-4^)	0.002	0.473 (NS)	0.211 (NS)	0.324 (NS)	-0.505 (NS)
*C. elegantior*											
Old World	16	3	4	0.442	0.4833	9 × 10^-4^ (3.6 × 10^-4^)	0.002	-1.316 (NS)	-1.122 (NS)	-1.351 (NS)	-1.867 (NS)
New World	67	7	7	0.662	1.003	0.002 (2.7 × 10^-4^)	0.003	-0.791 (NS)	-0.420 (NS)	-0.638 (NS)	-1.614 (NS)
Mountain USA	62	4	5	0.608	0.739	0.001 (1.8 × 10^-4^)	0.002	-0.288 (NS)	-0.153 (NS)	-0.228 (NS)	-0.753 (NS)
Pacific	5	1	2	0.400	0.400	7.8 × 10^-4^ (4.6 × 10^-4^)	9.3 × 10^-4^	-0.816 (NS)	-0.816 (NS)	-0.771 (NS)	0.090 (NS)
All (l = 516)	83	13	11	0.760	2.139	4.2 × 10-3 (4.2 × 10^-4^)	0.005	-0.495 (NS)	-0.782 (NS)	-0.808 (NS)	-1.468 (NS)
*C*. *napus*											
Old World	8	0	1	0.000	0.000	0.000	0.000	ND	ND	ND	ND
New World	19	2	3	0.368	0.385	6.8 × 10^-4^ (2.5 × 10^-4^)	1.0 × 10^-3^	-0.777	-0.573	-0.720	-0.725 (NS)
Mountain USA	16	1	2	0.325	0.325	5.8 × 10^-4^ (2.2 × 10^-4^)	5.3 × 10-^4^	0.155 (NS)	0.688 (NS)	0.627 (NS)	0.551 (NS)
Pacific USA	3	1	2	0.667	0.666	1.1 × 10^-3^ (5.6 × 10^-4^)	0.002	ND	ND	ND	0.201 (NS)
All (l = 564)	27	2	3	0.271	0.279	5.0 × 10^-4^ (2.0 × 10^-4^)	9.2 × 10^-4^	-0.977 (NS)	-0.702 (NS)	-0.898 (NS)	-1.088 (NS)

l, nucleotide sequence length; n, sample size; s, number of segregating sites; h, haplotypes; hd, haplotype diversity; k, average number of nucleotide pair wise differences; π, number of nucleotide differences per site (SD = standard deviation of π); θw, Watterson's estimate of θ per site; ND, not determined because no polymorphism was found; NS, non-significant; **, P < 0.02.

### Tests of neutrality and population subdivision

The neutrality tests performed for the whole-species data sampling had non-significant values; therefore, the equilibrium model of neutral evolution could not be rejected. Only in *C*. *arcuatorum *were significant values detected for Fu and Li's D*, and Fu and Li's F* tests, suggesting background selection for the whole sample (Table 1). The geographical distributions and frequency of the haplotypes are given in Additional File 1. With regard to *C*. *arcuatorum*, haplotype H1 (I) was only found in those samples obtained from Costa Rica (Jardín de Dota and Parque Prusia), while haplotypes H2 (II) and H3 (III) are specific to California (Mendocino and Del Norte Counties, respectively). The most frequent haplotype (H4, IV; 12 population samples) is distributed throughout the Old World (Germany, France, Italy, Sweden) and also occurs in the New World (Wyoming), see population samples IB19870107 and IB19870239. The single haplotype H5 (IV) is restricted to the Old World. In *C*. *aureofulvus*, haplotypes H1 and H2 are restricted to the Old World (Austria, France, Germany, Sweden), whereas haplotypes H4 and H5 were found in the New World (Washington State) population samples. Haplotype H3 was found in population samples from the Pacific Northwest (Washington State) and Mountain USA (Colorado and Wyoming). In *C*. *elegantior*, H7 (V) and haplotypes H1 (VII) were predominant in the New World (Wyoming) and the Old World (Austria, France, Germany and Switzerland), respectively. Haplotypes H2, H3 and H4 are restricted to Old World and haplotypes H5, H6 (VI), H8, H9, H10 and H11 were observed only in those population samples obtained from Old World. With regard to *C. napus*, the predominant haplotype H1 was shared by different taxa in the New World (Pacific: Oregon and Washington State, and Mountain USA: Wyoming and Colorado) and the Old World (Germany, Norway, Sweden). Haplotype H2 was found in the Wyoming populations and haplotype H3 is represented by a single population sample (JFA 10070) from the Pacific (Washington State).

Hudson's tests were carried out to estimate population genetic structures within and among population samples from each area (Table 2). Analyses of populations showed significant differentiation between Costa Rica/Pacific USA (California) and Mountain USA (Wyoming)/Europe population samples of *C*. *arcuatorum *(*P *< 0.001, K_ST _= 0.6540, K_S _= 5.0769, K_T _= 14.6769); between Europe/Pacific USA (Washington State, Oregon) and Mountain USA (Wyoming, Colorado) population samples of *C*. *elegantior *(*P *< 0.001, K_ST _= 0.0833, K_S _= 3.1645, K_T _= 3.4522) and between Europe/Pacific USA (Washington State) and Mountain USA (Wyoming, Colorado) population samples of *C*. *aureofulvus *(*P *= 0.001, K_ST _= 0.3183, K_S _= 1.4166, K_T _= 2.0784). Non-significant genetic differentiation between Europe/Pacific (Washington State, Oregon) and Mountain USA (Wyoming, Colorado) was found for *C. napus *population samples (*P *= 0.2240, K_ST _= 0.0518, K_S _= 0.3998, K_T _= 0.4216). Alternatively, significant *P *values (*P *< 0.001) for Hudson's tests indicated that New World and Old Wold population samples of *C*. *arcuatorum *(K_ST _= 0.4619, K_S _= 7.8968, K_T _= 14.6769), *C*. *aureofulvus *(K_ST _= 0.3731, K_S _= 1.3027, K_T _= 2.0784) and *C*. *elegantior *(K_ST _= 0.3927, K_S _= 2.0963, K_T _= 3.4522) were genetically differentiated, while New World and Old World macro-populations of *C. napus *(*P *= 0.6840, K_ST _= -0.0251, K_S _= 0.4322, K_T _= 0.4216) were not significantly differentiated from each other.

**Table 2:** Population subdivision in calochroid taxa inferred from Hudson's test statistics Ks (upper right matrix) and Kst (lower left matrix)

	Old World	Mountain USA	Pacific USA	Costa Rica
*C. arcuatorum*				
Old World		0.1818 (NS)	3.7041***	0.1636**
Mountain USA	-0.1818 (NS)		7.6666**	0.0000 (NS)
Pacific USA	0.7511***	0.4025**		6.8148*
Costa Rica	0.9754**	1.0000 (NS)	0.3185*	
*C*. *aureofulvus*				
Old World		0.5714*	1.3399***	
Mountain USA	0.3949*		1.8888 (NS)	
Pacific USA	0.4044***	-0.1052 (NS)		
*C. elegantior*				
Old World		1.7739***	0.6607***	
Mountain USA	0.4581***		1.9434***	
Pacific USA	0.7411***	0.1880***		
*C*. *napus*				
Old World		0.3908 (NS)	0.0952 (NS)	
Mountain USA	-0.0081 (NS)		0.5655 (NS)	
Pacific USA	0.4761 (NS)	0.0329 (NS)		

Significance, including incompatible sites and recombination blocks, was evaluated by performing 1000 permutations using SNAP Map. NS, non-significant; *0.01 ≤ P < 0.05; **0.001 ≤ P < 0.01; ***P < 0.001.

### Network analyses

**Figure 4:**
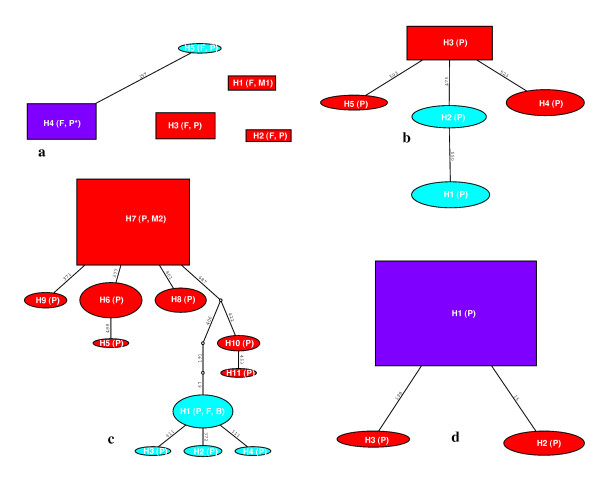
**TCS haplotype networks based on non-recombining ITS sequences**. **a**. *C. arcuatorum*. **b**. *C. aureofulvus*. **c**. *C*. *elegantior*. **d**. *C. napus*. Parsimony probabilities were set at 95%. Sizes of circular and rectangular areas are proportional to the number of individuals with that haplotype. Haplotypes with a blue background are from the Old World and those with red backgrounds are from the New World. Haplotypes comprising New and Old World populations are indicated by lilac backgrounds. Distributions of ecological and morphological features are abbreviated as follows: F, Fagaceae; P*, Pinaceae (pure stand in Wyoming); P, Pinaceae; B, Betulaceae, M1, populations of *C*. *arcuatorum *with larger spores; M2, some populations of *C*. *elegantior *with sulphur yellow veil and lacking of veil patches on the pileus surface.

To clarify the phylogenetic relationship among haplotypes within each species, networks were constructed using haplotypes from ITS sequences with non-recombining blocks (Figure 4a-d). Nested clade analyses for *C*. *arcuatorum *resulted in one network with two connected Old World haplotypes (H4 and H5) and three single unconnected New World haplotypes (H1, H2 and H3) (Figure 4a). Analyses of *C*. *aureofulvus *(Figure 4b) yielded a network comprising five haplotypes: haplotypes H3, H4 and H5 were scattered throughout the New World, whereas haplotypes H1 and H2 were restricted to the Old World. The haplotypes H1, H2, H4 and H5 were derived from haplotype H3. Similarly, in *C. elegantior*, haplotypes were contained in a single network where haplotype H7 has an ancestral position to the other haplotypes and the Old World haplotypes H1, H2, H3 and H4 grouped together (Figure 4c). The Old World haplotypes H1, H2, H3 and H4 appear to be more related to the Pacific haplotypes H10 (Oregon and Washington State) and H11 (Washington State). In *C. napus*, haplotype H1 is ancestral to the other haplotypes and was shared by both New World and Old World populations, whereas the haplotypes H2 and H3 were restricted to Mountain USA (Wyoming) and Pacific (Washington State) in the New World, respectively (Figure 4d).

### Migration analyses

The four species showed different histories of recombination: two distinct recombination blocks were detected in *C*. *arcuatorum*, four in *C*. *aureofulvus*, nine in *C*. *elegantior *and one in *C. napus*, respectively. There was no evidence of recombination within each of these blocks. Migration parameter estimates, including population mutation rate, time of divergence and direction of migration estimates between New World and Old World macro-population samples, are shown in Additional File 2. These analyses showed that migrations between New World and Old World macro-population samples within *C*. *arcuatorum *and *C*. *aureofulvus *were approximately equal in both directions and that these have occurred at low rates. While gene flow estimates in *C*. *elegantior *indicate that migration appeared to be slightly higher (with overlapping confidence intervals) from the New World to the Old World (m_1 _= 0.34) rather than *vice versa *(m_2 _= 0.16).

### Genealogical analyses

**Figure 5:**
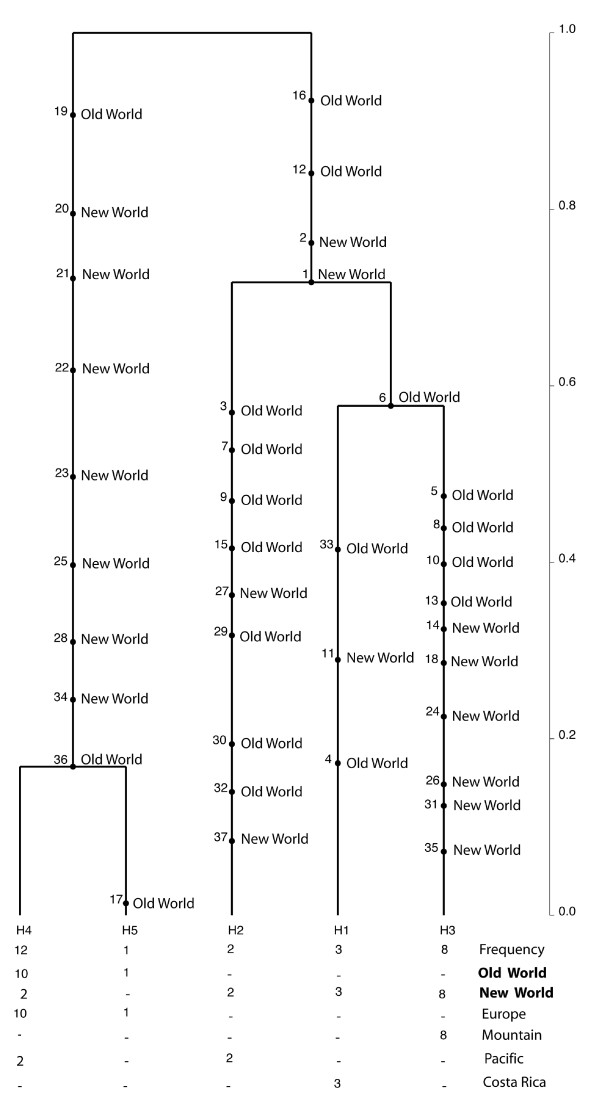
**Coalescent-based genealogy of the internal transcribed spacers (ITS1 and ITS2), showing the distribution mutations for New World (CR = Costa Rica; Pacific, CA = California; Mountain USA, WY = Wyoming) and Old World (EU = Europe) populations of *C. arcuatorum *performed using Genetree**. The inferred genealogy is a rooted genealogy based on five million simulations of the coalescent. Simulations were carried out assuming a constant population size. Maximum likelihood estimates of the tree with the highest root probability (likelihood = 1.16 × 10^-18^) and standard deviation (SD = 9.53 × 10^-16^) showing the distribution of mutations. The direction of divergence is from the top of the genealogy (oldest = past) to bottom (youngest = present); coalescence is from the bottom (present) to the top (past). The numbers below each branch indicate the corresponding haplotype and its frequency in each macro-population and sampling area. The time scale is in coalescent units of effective population size.

**Figure 6:**
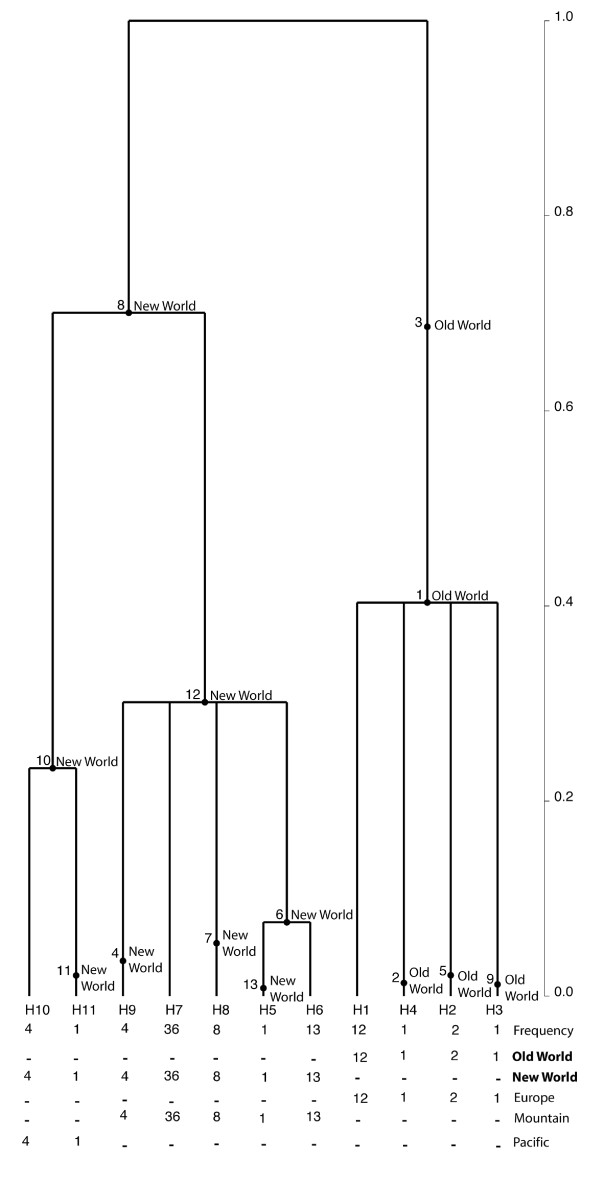
**Coalescent-based genealogy of the internal transcribed spacers (ITS1 and ITS2), showing the distribution mutations for New World (Pacific, WA = Washington State, OR = Oregon; Mountain USA, WY = Wyoming) and Old World (EU = Europe) populations of *C*. *elegantior *performed using Genetree**. The inferred genealogy is a rooted genealogy based on five million simulations of the coalescent. Simulations were carried out assuming a constant population size. Maximum likelihood estimates of the tree with the highest root probability (likelihood = 2.55 × 10^-15^) and standard deviation (SD = 4.68 × 10^-13^) showing the distribution of mutations. The direction of divergence is from the top of the genealogy (oldest = past) to bottom (youngest = present); coalescence is from the bottom (present) to the top (past). The numbers below each branch indicate the corresponding haplotype and its frequency in each macro-population and sampling area. The time scale is in coalescent units of effective population size.

**Figure 7:**
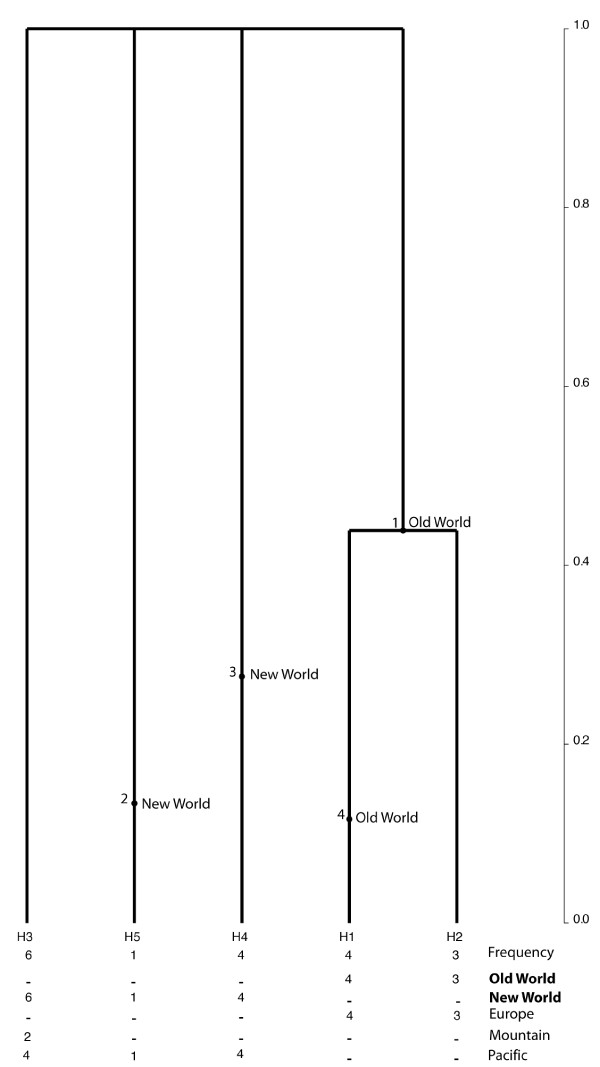
**Coalescent-based genealogy of the internal transcribed spacers (ITS1 and ITS2), showing the distribution mutations for New World (Pacific, WA = Washington State; Mountain USA, WY = Wyoming, CO = Colorado) and Old World (EU = Europe) populations of *C. aureofulvus *performed using Genetree**. The inferred genealogy is based on five million simulations of the coalescent. Simulations were carried out assuming a constant population size. Maximum likelihood estimates of the tree with the highest root probability (likelihood = 1.75 × 10^-8^) and standard deviation (SD = 1.61 × 10^-6^) showing the distribution of mutations. The direction of divergence is from the top of the genealogy (oldest = past) to bottom (youngest = present); coalescence is from the bottom (present) to the top (past). The numbers below each branch indicate the corresponding haplotype and its frequency in each macro-population and sampling area. The time scale is in coalescent units of effective population size.

Coalescent genealogies for *C*. *arcuatorum *and *C*. *elegantior *showed that two ancestral lineages gave rise to the extant New and Old World haplotypes (Figures 5 and 6). Genealogies inferred for *C*. *aureofulvus *(Figure 7) and *C. napus *(data not shown) did not have enough genetic polymorphisms to resolve transoceanic disjunction events. In *C*. *arcuatorum*, a widely distributed (New World (Wyoming) - Old World (Europe)) ancestral macro-population could be the origin of extant haplotypes. In *C*. *elegantior*, an ancestral macro-population diverged at 0.7 into two distinct lineages in the New World, whereas Old World haplotypes diverged at 0.4, respectively. The oldest mutations of both New World and Old World macro-populations of *C*. *arcuatorum *have a mean age of 0.9, while the *C*. *elegantior *macro-populations have a mean age of approximately 0.7. In *C*. *arcuatorum*, extant Old World haplotypes are younger than New World haplotypes, T < 0.2 and T > 0.5, respectively. In *C*. *elegantior*, New World haplotype deep divergence was older (T > 0.6) than New World haplotypes (T = 0.4), but extant New World haplotypes are descendents from more recent lineages.

### Basidioma phenotypical features

Detailed descriptions of the macro- and microscopical structures for each species are given in Additional File 3. The most relevant phenotypical features are summarised below.

#### (i) Basidioma colour

**Figure 8:**
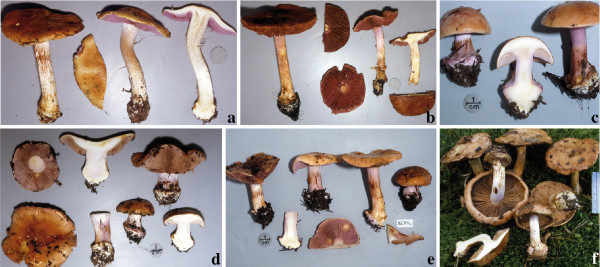
**Basidioma morphology and colouration within *C*. *arcuatorum***. **a**. Costa Rican population JFA 12039. **b**. Costa Rican population JFA 12061. **c**. Californian (Mendocino) population IB19950686, **d**. Californian (Patricks Creek Campground) population JFA 11765. **e**. Californian (Patricks Creek Campground) population IB19950564. **f**. European population TUB 011403. Photos c and e courtesy of M. Moser.

**Figure 9:**
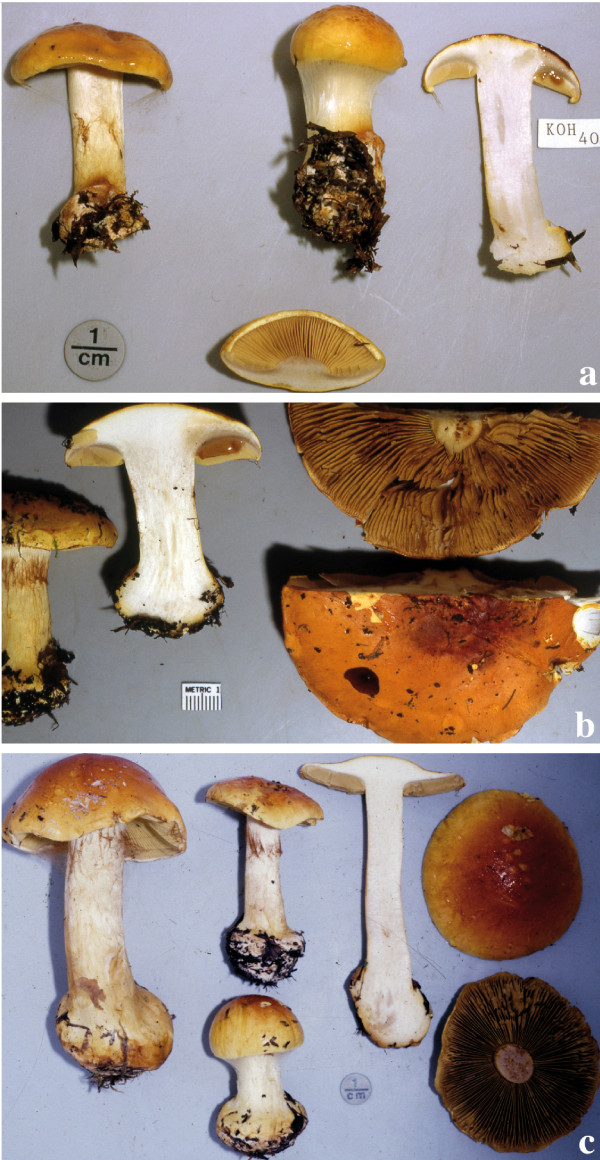
**Basidioma morphology and colouration of *C*. *aureofulvus***. **a**. Washington State population IB19890428. **b**. Colorado population JFA 12428. **c**. Swedish population IB19850209. Photos a and c courtesy of M. Moser.

**Figure 10:**
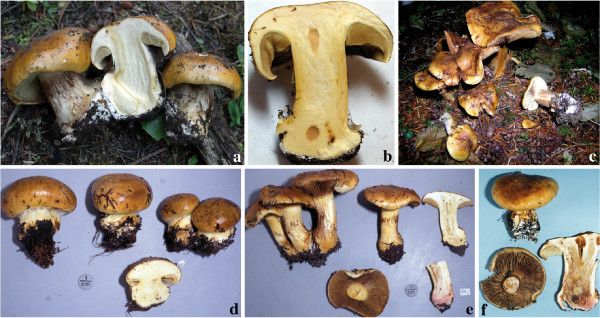
**Basidioma morphology and colouration within *C*. *elegantior***. **a**. Washington State (Table Mountain) population JFA 13287. **b**. Washington State (Table Mountain) population JFA 13287, a basidioma showing KOH reaction. **c**. Washington (Snohomish County) population TUB 019280. **d**. Wyoming (Teton National Forest) population of *C*. *elegantior *var. *americanus *IB19890059 (holotype). **e**. Wyoming (Teton National Forest) population IB19890189; NH_3 _is showed on stipe context. **f**. Austrian population IB19790599 (one basidioma shows a red wine KOH reaction). Photos d, e and f courtesy of M. Moser.

**Figure 11:**
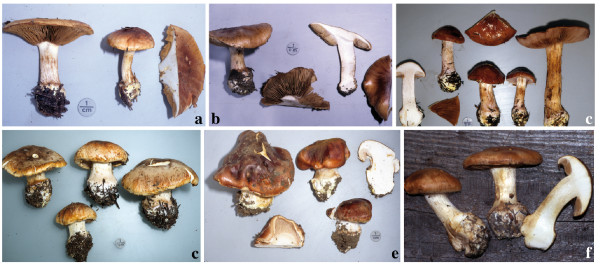
**Basidioma morphology and colouration within *C. napus***. **a**. Washington State (Rainy Pass) population IB19890479. **b**. Washington (Chelan County) population sample of *C*. *albobrunnoides *var. *violaceovelatus *IB19890186 (typus). **c**. Oregon (Lincoln County) population sample of *C*. *subpurpureophyllus *var. *sulphureovelatus *JFA 11723. **d**. Wyoming population (Shoshone National Forest) IB19970303. **e**. Wyoming (Shoshone National Forest) population IB19910237. **f**. European population TUB 019282. Photos a, b, d and e courtesy of M. Moser.

There was little or no detectable variation of basidiomata colour within *C*. *arcuatorum*, *C*. *aureofulvus *and *C*. *elegantior *(see Figures 8, 9 and 10). However, population samples of *C. napus*, showed considerable variation in colour of both lamellae and veil (Figure 11).

#### (ii) Basidioma size

Some variation in basidioma size was observed within *C*. *arcuatorum*, where populations from Costa Rica and some from Europe presented somewhat slender basidiomata in comparison with other population samples (Figure 8).

#### (iii) Colour reaction of dried specimens

**Figure 12:**
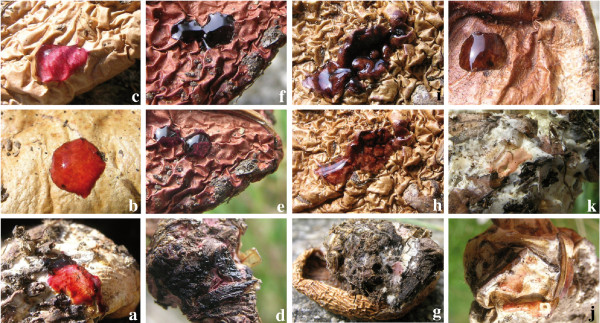
**Colour change reactions with KOH 40% mycelia at the stipe bulb and on pileus surface of dried specimens**. **a - c: ***C. arcuatorum*: **a**. Blood colour on mycelia in IB19870239. **b**. Blood colour on pileus surface in IB19870239. **c**. Pink on pileus surface in TUB 019283. **d - f: ***C. aureofulvus*: **d**. Black on mycelia in IB19890428. **e**. Purple on pileus surface in IB19930612. **f**. Reaction in (**e**), turning black after some minutes. **g - i: ***C*. *elegantior *var. *americanus *IB198959 holotype: **g**. Wine red on mycelia. **h**. Intense wine red on pileus surface. **i**. Reaction in (**g**), after a while becoming dark wine red. **j**. Pink with orange tinge on mycelia in *C*. *napus *TUB 019282. **k - l**: *C*. *albobrunnoides*. **k**. Pink with orange tinge on mycelia in *C*. *albobrunnoides *IB19890298. **l**. Dark red-brown on pileus surface in *C*. *albobrunnoides *IB19970303. Note: photographs were taken in natural daylight.

A pink to pinkish orange colour reaction with 40% KOH on both pileus surface and mycelia at the stipe base characterised the basidiomata of *C*. *arcuatorum *populations. Population samples of *C. napus *yielded a greyish to dark red brown colour reaction on the pileus surface, whereas the mycelia showed a pale pink to intense pink colour reaction. All population samples in *C*. *aureofulvus *had a black reaction on both pileus surface and mycelia on the stipe bulb, while in *C*. *elegantior*, both the pileus surface and mycelia turned wine red (Figure 12).

## Discussion

The markedly high species diversity within the genus *Cortinarius *together with their obligate ectomycorrhizal status makes the factors that have driven their speciation of particular interest to a broad spectrum of biologists. In addition, the occurrence of widespread taxa within *Cortinarius *raises intriguing questions about the origins of transoceanic disjunct species and the influence of the historical events that have created contemporary geographical distributions.

### Phylogeographical structure and historical demography of *Calochroi *species

While it is difficult to determine the origin and migration routes of ectomycorrhizal fungi between North America, Europe and Asia, one can gain some insight into the question by looking at host migration patterns and assuming co-migration of plant and fungus. Genetic data reveal considerable divergence between populations across the core range of distribution of *C*. *arcuatorum *and *C. elegantior *in the New World (Figure 4a, c; Table 2). Since the divergent ancestral lineages were found in the New World, we postulate this area as the centre of origin for *C*. *arcuatorum *and *C*. *elegantior*, and that Old World populations are the result of relatively recent demographic expansion (Figures 4a, c, 5 and 6). Migration with host plants [28] through Beringia is one of the most plausible hypotheses to explain the disjunct population distributions between the New World and Old World, but migrations through the North Atlantic land bridge [29] or via long-distance dispersal [30], though a less likely explanation cannot be completely ruled out. Genetic breaks between *C*. *arcuatorum *populations within the New World have probably been triggered by paleogeographic and paleoclimatic events, e.g. the rise of the Sierra Nevada and other mountains followed by desertification of the Great Basin, or other events such as Pliestocene glaciation [30,31]. The European populations of *C. arcuatorum *and most of the North American populations occur mainly with trees of the family Fagaceae, with the exception of the population samples in Wyoming, where this species occurs with conifers. These known population locations are currently geographically isolated from members of the Fagaceae; however, it is likely that *Picea *and/or *Tsuga *were sympatric with *Quercus *in the past [32], allowing for the possibility of a host switch from *Quercus *to a conifer host.

The coalescent-based analysis suggests that extant populations diverged from a quite recent common ancestor early in the evolution of *C*. *elegantior *with little gene flow among disjunt populations (Figure 6; Additional File 2). Despite this, very little morphological change has evolved between New World and Old World lineages. Hence, the close relatedness of Pacific and European haplotypes of *C*. *elegantior *could indicate an expansion northward along the western side of the mountains through the Bering Land Bridge, which resulted in the extant populations in New World (Figure 4c). This evolutionary scenario is compatible with the migration of *Tricholoma *(matsutake) between western North America and eastern Asia, as postulated by Chapela & Garbelotto [8]. The phylogeographical patterns observed in *C*. *elegantior *support the hypothesis that the current distributions of populations are closely associated with the historical events of their host plants. Similarly, conifers are the primary hosts for populations of *C. aureofulvus *and for *C. napus*. In most parts of their ranges, these *Cortinarius *species are sympatric or allopatric, although their frequency of occurrence varies, with *C. aureofulvus *being the least commonly encountered, *C. napus *being moderately frequent, and *C. elegantior *populations being the most frequently encountered, especially at higher elevations with *Picea*. Populations of *C. elegantior *and *C. napus *are most frequently encountered in subalpine conifer forests; however, they also occur in more mesic mid-elevations and coastal sites. *Picea *is currently a main host plant genus for *C. aureofulvus*, *C. elegantior *and *C. napus *in western North America; this has probably been the case for an extended period of time, with a shift in abundance of *Picea *from eastern to western North America several times over the last 21,000 years [32]; however, *Abies*, *Pinus *and *Tsuga *are also potential hosts for these species. Douglas fir, another possible host for these species, appeared in western North America in the mid-Pleistocene [33]. Recent phylogenetic studies of *Picea *indicate that it originated in North America (*P. breweriana *and *P. sitchensis *as basal taxa) and that the present distribution of *Picea *could be a result of two dispersal events, one from North America to Asia by the Bering Land Bridge [34], and a second from Asia to Europe. Most of the northeastern Asian species and the European *P. abies *could have arisen from a recent radiation [35]. The literature also notes that the earliest (Paleocene) fossil of *Picea *is from Montana and that it is well represented in the Eocene in western North America, but was not common in Asia until the Oligocene and not present in Europe until the Pliocene. Therefore, it is fully possible that populations within *C*. *aureofulvus*, *C. elegantior *and *C. napus *have long been associated with *Picea *and could have migrated with this and possible other host plants during glacial and interglacial periods for millions of years. The findings presented here, in conjunction with those of Dentinger *et al*. [4] and Wu *et al*. [7] strongly support the key role of Beringia in the phylogeographic processes leading to speciation and intraspecific population structures in the Northern Hemisphere. Subsequently, the comigration with their associated phanerogams [28,35] through e.g. short-distance spore dispersal may be an important means explaining the expansion of these fungi from Asia to Europe.

We cannot estimate times for the divergence of disjunct populations of *Calochroi *with certainty, owing to a lack of fossil records. However, it is plausible to hypothesise that they co-migrated with their host plants, and that the lineages may have subsequently diverged as a result of fragmentation and geographic isolation of ancestral populations due to geologic and subsequent climatic changes. However, each species has a somewhat different biogeographical history that likely has been influenced by different biotic and abiotic factors, such as dispersal potential and host (phanerogam) history. The occurrence of these taxa in Asia has not been documented to date. Consequently further sampling across this region is necessary in order to better understand their historical distributions across the Northern Hemisphere. The recent work on porcini mushrooms [4] supports the idea of a potential link between European, Asian and North American species in the section *Calochroi*.

### ITS sequences and phenotype as identification tools for *Cortinarius *species with wider ranges of geographical distribution; utility and limitations

Nested clade, demographic and coalescent-based analyses based on the ITS region of *C*. *arcuatorum*, *C*. *aureofulvus*, *C*. *elegantior *and *C. napus *allowed us to infer their evolutionary histories. Overall, *C*. *arcuatorum *and *C*. *elegantior *exhibited geographically structured haplotypes, with evidence for a more ancient population expansion. These findings agree with those of Geml *et al*. [9,11], who observed a similar population structure in the widespread fly agaric *Amanita muscaria*. Interestingly, geographically structured populations within *C*. *arcuatorum *and *C. elegantior *were often accompanied by some divergence in basidioma colouration and size in the absence of any described species (see Additional File 3). By contrast, some populations within *C. arcuatorum *and *C*. *aureofulvus *with disjunct distributions in North America and Europe exhibited little morphological differentiation and had identical to relatively low levels of ITS sequence divergence (identical to 0.3% (2 bp) divergence). Two hypotheses might explain this low degree of nucleotide variation. First, these taxa might be relatively young, having recently spread across their ranges, and, accordingly, having had insufficient time to accumulate ITS sequence divergence. An alternative hypothesis is that these results could be a consequence of low mutation rates at the locus studied. Although some differences in colouration of the basidiomata were found between western populations of *C. albobrunnoides *var. *albobrunnoides*, *C. albobrunnoides *var. *violaceovelatus *and *C. subpurpureophyllus *var. *sulphureovelatus *and European populations of *C. napus*, these differences were not sufficient to consider them as separate taxa. This suggests that variation in colouration alone might not necessarily reflect different species or subspecies. For example, violet pigments in the basidiomata are highly labile because they are sensitive to solar radiation and the age of the basidiomata. Therefore, it is evident that it is important to do careful fieldwork and to consider the variation of macroscopical and ecological features before they are used for taxonomic purposes, especially characters related to colouration. According to our analyses, colour reactions with KOH are relatively stable among populations, whereas ITS sequences, in certain instances, provide several genetic markers to distinguish species as well as population structure within the section *Calochroi*.

## Conclusions

Based on variations in ITS DNA polymorphism, this study revealed different evolutionary histories for New World and Old World populations within *Cortinarius arcuatorum*, *C. aureofulvus*, *C*. *elegantior *and *C. napus*. Nested clade, demographic and coalescent-based analyses provide powerful tools for assessing the effect of historical and contemporary events of the geographic distributions of these species, proposed as follows: (i) an ancestral population of *C. arcuatorum *putatively with a Wyoming-Europe distribution evolved into at least three distinct lineages in association with a variety of fagaceous and coniferous trees in the New World; (ii) two divergent lineages in *C*. *elegantior *gave rise to New World and Old World haplotypes that occur almost exclusively in association with *Picea *(apart from one Washington State population in *Tsuga*/*Abies *forest); (iii) low genetic diversity among New World and Old World populations of *C. aureofulvus*; iv) low genetic diversity among New World and Old World populations of *C. napus*. The results for *C. aureofulvus *and *C. napus *could be due to recent demographic expansion, but the origin of such transoceanic disjunct distributions remains unclear. The scenario of a spatial expansion through the Bering Land Bridge appears to be the most probable explanation for modern transoceanic disjunct distributions; however, it does not represent a complete explanation for the remarkably low degree of genetic differentiation between populations of these two species. Our study reveals patterns of species diversification restricted to the New World within *C. acuatorum *and *C. elegantior*, however, the relatively few morphological innovations found among allopatric populations suggest that diversification events may have been driven by ecological opportunities afforded by the shift to new host species, edaphic and climate conditions. The morphological and genetic data described here support a single species *C. napus *(to include *C. albobrunnoides *var. *albobrunnoides*, *C. albobrunnoides *var. *violaceovelatus *and *C. subpurpureophyllus *var. *sulphureovelatus*) with transoceanic distribution. Similarly, *C. aureofulvus *shows disjunct distributions in New World and Old World. Colour reactions with KOH represent a powerful tool for recognising these *Calochroi *species, whereas features related mainly to veil and lamellae colouration displayed high levels of homoplasy and phenotypic plasticity. Interpretations of the population structures suggest that host tree history as influenced by past events (glaciation, mountain building, and associated factors), was one of the driving forces that shaped the modern distributions of *Calochroi *species. Finally, our work shows the need for further studies that focus on careful fieldwork in the context of the variation of both morphology and ecology, as well as the use of more genetic information, to draw more complete models of population histories in these and other species of *Cortinarius*.

### Taxonomic implications

See Additional File 3 for information of the following taxonomic novelties:

***Cortinarius elegantio-montanus ***Garnica & Ammirati (= *Cortinarius elegantior *(Fr.) Fr. var. *americanus *M.M. Moser & McKnight, 1995)**, new synonym **and **new status**.

***Cortinarius elegantio-occidentalis ***Garnica & Ammirati, **new species **- allied to *Cortinarius elegantior *(Fr.) Fr., 1838.

***Cortinarius fulvo-arcuatorum ***Garnica & Ammirati, **new species **- allied to *Cortinarius arcuatorum *Rob. Henry, 1939.

***Cortinarius jardinensis ***Garnica, Ammirati & Halling, **new species **- allied to *Cortinarius arcuatorum *Rob. Henry, 1939.

***Cortinarius lilaciotinctus ***Garnica & Ammirati, **new species **- allied to *Cortinarius arcuatorum *Rob. Henry, 1939.

**Cortinarius napus **Fr. 1838 (= Cortinarius albobrunnoides var. albobrunnoides M.M. Moser & McKnight, 1995; Cortinarius albobrunnoides M.M. Moser & K. McKnight var. violaceovelatus M.M. Moser & J. Ammirati, 1996; and Cortinarius subpurpureophyllus A.H. Smith var. sulphureovelatus M.M. Moser, 2000), **new synonym**.

The authors declare that they have no competing interests.

## Methods

### Population sampling

We collected population samples within *C*. *arcuatorum *Rob. Henry, *C. aureofulvus *M.M. Moser, *C*. *elegantior *(Fr.) Fr. var. *elegantior *(including *C*. *elegantior *(Fr.) Fr. var. *americanus *M.M. Moser & McKnight) and *C. napus *Fr. (including *C. albobrunnoides *M.M. Moser & McKnight, *C. albobrunnoides *var. *violaceovelatus *M.M. Moser & Ammirati, and *C. subpurpureophyllus *A.H. Sm. var. *sulphureovelatus *M.M. Moser) groups, following their distribution ranges in Europe, Central America and western North America. The following specimens were initially used for DNA sequencing: 1 collection of *C. albobrunnoides *from the University of Washington Herbarium (WTU) and 13 from Innsbruck Herbarium (IB) and 8 collections of *C. arcuatorum *(including two named *C*. cf. *sodagnitus *IB19870239 and IB19870107); 36 collections of *C. elegantior *(including the type material of *C*. *elegantior *var. *americanus *IB19890059) from Europe and North America deposited at (IB) and 4 from WTU; 14 collections of *C. aureofulvus *and 7 collections of *C. napus *from IB. An attempt was made to sample from throughout the distribution range of these species, although sampling was dictated by the availability of recently collected and accurately documented herbarium specimens. These four taxa were selected because they represent a diverse set of species/populations, each with a monophyletic lineage displaying allopatric distributions in Europe and North America, as previously revealed in research by the authors [14]. In order to incorporate as much genetic information as possible from a wide range of geographical locations for these species, sequences were obtained from GenBank http://www.ncbi.nlm.nih.gov/ and UNITE http://unite.ut.ee/ databases. Details of the population samples used for this study and the geographic location of the sampling sites are provided in Additional File 4.

### DNA extraction, amplification, sequencing and sequence editing

Total genomic DNA was extracted from dried lamella fragments using the DNAeasy Plant Mini Kit (Qiagen, Hilden, Germany) following the standard protocol. The internal transcribed spacers (ITS1, ITS2), the 5.8 S ribosomal subunit and the D1/D2 regions of the nucLSU were amplified with the primer combination ITS1F (5'-CTTGGTCATTTAGAGGAAGTAA-3') [36]/NL4 (5'-GGTCCGTGTTTCAAGACGG-3') [37]. Subsequently, those specimens that did not give any amplification products were amplified with the primer combinations ITS1F/ITS4 (5'-TCCTCCGCTTATTGATATGC-3') [38] or ITS1 (5'-TCCGTAGGTGAACCTGCGG -3') [38]/ITS4 and 5.8SR (5'-TCGATGAAGAACGCAGCG-3')/LR3 (5'-CCGTGTTTCAAGACGGG-3') [39] or 5.8SR/NL4. Amplifications of ITS1, ITS2, the 5.8S ribosomal subunit and the D1/D2 regions of the nucLSU were carried out using a touch-down program with the following conditions: initial denaturation at 94°C for 3 min; 10 cycles with temperatures ranging from 60°C in the first cycle to 51°C, each cycle decreasing by 1°C; 25 cycles with an annealing temperature of 50°C, each cycle consisted of an annealing step of 0.5 min; an elongation step of 72°C for 1 min and a denaturation step of 94°C for 0.5 min and a final elongation phase at 72°C for 7 min. Alternatively, amplifications of the ITS region for those specimens with negative results using Taq DNA polymerase (Invitrogen Corporation, Carlsbad, CA, USA) were carried out using 25 μL Phusion™ High-Fidelity DNA polymerase-mediated reactions (Finnzymes Oy, Keilaranta, Finland) with an initial heating of 95°C for 60 seconds, followed by 35 cycles of denaturation at 94°C; 30 seconds, annealing at 50°C-55°C; 30 seconds and extension at 72°C; and 45-90 seconds, followed by a final extension of 72°C for 10 minutes. Amplification products were electrophoresed in a 0.7% agarose gel and stained with ethidium bromide for visualization of the bands. PCR products were cleaned using ExoSAP-IT^® ^(USB Corporation, Cleveland, OH, USA) reagent diluted 1: 20 according to the manufacturer's instructions. Both DNA strands were cycle-sequenced with the amplification primers and, in some cases, internal primers as indicated in Garnica *et al*. [40]. Clean PCR products were sequenced in both directions with a 1: 6 diluted dye terminator sequencing kit (Big Dye 3.1; Applied Biosystems, Foster City, CA, USA) on an ABI Prism 3130 × l Genetic Analyzer (Applied Biosystems). Unpublished molecular data from our lab indicate non-sequence divergence among individuals within the same collection; therefore, we sequenced only one individual per collection. Forward and reverse sequence fragments were assembled and edited using Sequencher version 4.1 (Gene Codes Corporation, Ann Arbor, MI, USA).

### Sequence identity, alignments, phylogenetic placement and variability

To detect potential contaminant sequences, we first screened our sequences against those available in the GenBank database http://www.ncbi.nlm.nih.govhttp:///[41]) using the Basic Local Alignment Search Tool (BLAST). To establish the phylogenetic placement of the newly generated ITS sequences and those from Garnica *et al*. [14], we automatically aligned them in MAFFT v5.7 [42] with the E-INS-i option. Our data matrix, which included 637 sequences and 739 nucleotide positions, was analysed by ML inference as implemented in RAxML version 7.0.3 [43]. The best-known likelihood tree under the GTRMIX model of nucleotide substitution was computed from 100 runs starting from distinct randomised maximum parsimony starting trees. A total of 1000 non-parametric bootstrap replicates [44] were run on the original alignment. Graphical processing of the trees with best likelihood and bootstrapping were generated using TreeViewPPC version 1.6.6 [45] and PAUP* 4.0b10 [46]. Subsequently, sequences for each taxon were aligned separately in MAFFT. Within each taxon set, all polymorphic sites were rechecked from the chromatograms. Isolates from fungal specimens are *n *+ *n *and therefore provide ambiguous haplotype data for heterozygotes. Heterozygous sites appear as two coincident peaks at the same site in the forward and reverse sequence chromatograms. To resolve the haplotype structure within the heterozygote, we used Clark's haplotype subtraction algorithm [47]. The DNA sequences newly generated in this study were submitted to GenBank (accession numbers GU363452-GU363497).

All sequences within one species were collapsed into haplotypes removing indels and excluding infinite-site violations using Map as implemented in SNAP Workbench [48,49]. A site-incompatibility matrix was calculated to assess compatibility among all variable characters, with incompatible characters being subsequently removed. Sites are called compatible if there is a phylogeny which both can evolve on without homoplasy. Two or more variable sites showing an identical pattern of compatibility are considered to form a recombination block [50]. When more than one recombination blocks were found, we used RecMin [51] as implemented in SNAP Workbench to estimate the minimum number of recombination events. Conversely, all sites being compatible with each other were considered as evidence for no recombination. Sequence polymorphism (haplotype h and nucleotide π diversities) was estimated using DnaSP version 5 [52]. Estimates were calculated for the entire dataset comprising all sequences of one species, as well as for each subpopulation sampled.

### Neutrality tests and population subdivision

Identical ITS sequences were collapsed into haplotypes using SNAP Map [49] after excluding indels and infinite-site violations. Site compatibility matrices were generated from each haplotype datasets using SNAP Clade and Matrix [53]. Sequence variations were tested for deviations from neutrality by using Tajima's D [54], Fu and Li's D* and F* [55], and Fu's Fs [56] statistics with DNASP v 5.00.07 [52]. Tests of neutrality assume a constant population size, no recombination and no migration. The D* test by Fu and Li is based on assessing selection by counting external (recent) and internal (ancient) mutations in the genealogy and comparing the values to their expectations under the assumption of neutral evolution. Significant Fu and Li's tests suggest background selection, while a significant Fu's Fs value indicates population growth and genetic hitchhiking. As small sample sizes and population subdivisions are known to limit the power of the neutrality tests [57,58], we subsequently tested our data for population subdivision. To test for population genetic differentiation, we used SNAP Map [49] to generate the appropriate sequence file, Septomatrix to convert the sequence file to a distance matrix, and Permtest [57] to test for geographic intraspecific subdivision among the different areas sampled, as implemented in SNAP Workbench [48]. Population genetic differentiation analyses were calculated according to the sampled areas in Central America (CR = Costa Rica), Pacific USA (WA = Washington State, OR = Oregon, CA = California), Mountain USA (WY = Wyoming, CO = Colorado) and Europe (EU). In order to increase sample sizes, populations were grouped into macro-populations with regard to the directions of migration hypothesized as follows: for *C. arcuatorum*, these were Costa Rica (CR) + Pacific (CA) + Mountain USA (WY); for *C. aureofulvus*, these were Pacific USA (WA) + Mountain USA (WY, CO)*; *for *C*. *elegantior*, Pacific (WA, OR) + Mountain USA (WY); for *C. napus*, Pacific USA (WA, OR) + Mountain USA (WY, CO). Each macro-population was compared against the EU macro-population. The main criteria used to define the macro-populations were: geographical location of the sampling sites, geological events and host tree histories. Alternatively, population samples were analysed as follows: for *C. arcuatorum *in Central America (CR) + Pacific (CA) macro-populations was compared against Mountain USA (WY, CO) + Europe (EU) macro-populations, whereas in *C. aureofulvus *and *C. elegantior *Pacific USA (WA, OR) macro-populations were compared against Mountain USA (WY) + Europe (EU) macro-populations. Hudson's test statistics were evaluated under the null hypothesis of no genetic differentiation applying, 1,000 permutations per species sample. For this purpose only, we used the non-reduced datasets containing the recombination blocks (see above), as recombination has been shown to improve the power of Hudson's test [57].

### Haplotype network analyses

We constructed haplotype networks by using the program TCS version 1.21 [59]. Parsimony probability was set at 95%; therefore, haplotypes with a probability of parsimony higher than 95% would be connected and those with a probability lower than 95% would be unlinked.

### Migration analysis

For those species showing significant population subdivision according to Hudson's test, we conducted migration analysis using the IM program [60]. As the program model assumes neutrality and no recombination, the reduced dataset that contained non-recombinant characters only was submitted to analysis. This program simultaneously infers multiple genealogical parameters like effective population sizes for ancestral (**θ_A_**) and modern (**θ_1 _**and **θ_2_**) populations, time since divergence between populations (t) and migration rates (m_1 _and m_2_) between two subpopulations using a Markov Chain Monte Carlo (MCMC) approach. We used chain lengths of 10,000,000 steps for *C*. *elegantior *dataset and 20,000,000 steps for *C*. *arcuatorum *and *C*. *aureofulvus *to guarantee a sufficiently large effective sample size (ESS), as well as a flat or presumably complete appearance of the posterior distribution curve. For each dataset, we executed a total of 15 independent simulations of the chain (starting from different random number seeds for each) and evaluated the estimated means of migration rates m_1 _and m_2_. Subsequently, a one-sided *t*-test was used to statistically assess the hypothesis of directional migration between the two subpopulations.

### Genealogical analyses

Ancestral intraspecific genealogies were inferred using Genetree Version 9 [61] in SNAP Workbench [48]. The genealogies with the highest root probability, ages of mutations, the times to the most recent common ancestor (TMRCA), and geographic distribution of the mutations were assessed from coalescent simulations. Coalescent simulations were carried out assuming an infinite-site model, constant size and population subdivision. As for the previous analyses, a reduced dataset was used that lacked incompatible and recombinant sites to meet the model assumptions. The backward migration matrix was set up by averaging the backward migration rate estimates obtained from the preceding IM simulations. Genealogical analyses for each species were estimated using 4,000,000 steps per coalescent simulation. For the most likely tree topology two independent runs using different starting seeds were executed, both resulting in the same topology.

### Distribution of phenotypic features among populations

To evaluate the distribution of morphological and macrochemical features of the basidiomata of the populations used in this study, we analysed the macro- and microscopical morphological structures in detail, as well as analysing the macrochemical reactions with KOH following standard procedures. Shape, measurement and colour of the microscopic features were obtained from dried specimens by mounting them in 3% KOH. Measurements of basidiospores (*n *= 51) are given as length and breadth, where the values in brackets represent the uncommon extremes. Basidiospore length/width quotient (Q), mean and standard deviation values were calculated.

In accordance with article 29 and 30A.2 of the International Code of Botanical Nomenclature, copies of this article are deposited at the following ten botanical and/or publicly accessible libraries: 1) Departamento de Botánica, Instituto de Biología U.N.A.M., Circuito exterior s/n, Cuidad Universitaria, Copilco, Coyoacán, Distrito Federal. C.P. 04510, A.P. 70-233 Mexico; 2) Marian Koshland Bioscience and Natural Resources Library, 2101 Valley Life Sciences Bldg. #6500, University of California, Berkeley, CA 94720-6500, USA; 3) Natural Sciences Library, Box 352900, University of Washington, Seattle, WA 98195, USA; 4) Serial and Electronic Resources, Washington State University Libraries, PC Box 645610, 100 Dairy Rd, Pullman, WA 99164-5610, USA; 5) Acquisitions, The LuEsther T. Mertz Library, The New York Botanical Garden, 2900 Southern Blvd, Bronx, NY 10458, USA; 6) Librarian, Canadian Forest Service, Natural Resources Canada, Pacific Forestry Centre Victoria, 506 West Burnside Road, BC Canada V8Z 1M5; 7) UBC Herbarium, Dept. of Botany, #3529-6270, University Blvd. Vancouver, B. C. Canada V6T 1Z4; 8) Plant Research Library, Wm. Saunders Bldg. #49, C.E.F., Otawa, ON, Canada; 9) Main Library, Herbarium, Royal Botanic Gardens, Kew, Surrey, TW9 3AB, UK; 10) Herbarium MSB, Ludwig-Maximilians-Universität München, Menzinger Straße 67, 80638 München, Germany; and 11) Herbarium Tubingense (TUB), University of Tübingen, Auf der Morgenstelle 1, D-74076 Tübingen, Germany.

## Authors' contributions

SG, JA and FO conceived the study design; SG generated the DNA sequences and wrote the manuscript; PS and SG performed population genetics analyses; macro- and microscopical descriptions of North American populations were made by JA; macroscopical descriptions of European populations were carried out by BO; SG studied the European collections microscopically. All authors read and approved the final manuscript.

## Supplementary Material

Additional File 1 **Identity of haplotypes of calochroid taxa inferred from ITS rDNA sequences**. Haplotype frequencies are shown in parentheses. In some cases, a single collection can carry two or more different haplotypes. Click here for file

Additional File 2 **Population mutation rate, effective sample size (ESS), time of divergence and direction of migration estimates within *C. arcuatorum, C. aureofulvus *and *C. elegantior *population samples**. The parameters are as follows: **θ_1_, θ_2 _**and **θ_A_**, are the mean population rates for the Old World, the New World and the ancestral population, respectively; t is the mean time of divergence of populations from a common ancestor; m_1 _represents the mean number of migrations into the Old World and m_2 _is the mean number of migrations into the New World. Numbers in parenthesis are standard deviations. Reliable estimation of the highest probability density (HPD) intervals could not be achieved, as the posterior distribution turned out to be incomplete in several cases. All values represent arithmetic means taken from 15 independent runs per species. Click here for file

Additional File 3 **Taxonomy**. Macroscopical descriptions are based on fresh material, whereas microscopical structures were analysed from dried specimens. Click here for file

Additional File 4 **Population samples used in this study and their respective voucher numbers, GenBank numbers, collection date, collection site, host tree(s), collector and determinator**. Holotype specimens are marked in bold. Herbarium abbreviations: IB = Herbarium Innsbruck, Austria; JFA = J. F. Ammirati material deposited at the Burke Museum, Herbarium, University of Washington Herbarium, Seattle, USA (WTU), Costa Rican National Biodiversity Institute (INB), and University of Costa Rica, San José (USJ); TUB = Herbarium Tübingense, University of Tübingen; S = Herbarium Stockholm, Sweden. Click here for file
