# Supplementary material for: Tracking the evolutionary history of Cortinarius species in section Calochroi, with transoceanic disjunct distributions
Source: BMC Evol Biol. 2011 Jul 19;11:213. doi: 10.1186/1471-2148-11-213 (PMC3161008; doi:10.1186/1471-2148-11-213)
Supplement: Additional File 1 — Identity of haplotypes of calochroid taxa inferred from ITS rDNA sequences. Haplotype frequencies are shown in parentheses. In some cases, a single collection can carry two or more different haplotypes. [file 1471-2148-11-213-S1.PDF]

Species	Haplotype (Frequency)	Collections	Population samples
C. arcuatorum	H1 (3)	JFA 12037, JFA 12039, JFA 12061	Costa Rica
	H2 (2)	JFA 11893, IB19950686	Pacific (California, Mendocino)
	H3 (8)	JFA 11766, JFA 11765, JFA 11803, IB19950564, IB19950596	Pacific (California, Del Norte Co.)
	H4 (12)	TUB 019283, TUB 019278, IB19870107, TUB 011403, TUB 011421, TUB 011447, IB19980286, PML5208, TSJ2000-083, TSJ2004-043, IB19870239, TUB 019279	Europe Mountain (Wyoming)
	H5 (1)	TUB 011403	Europe
C. aureofulvus	H1 (3)	TUB 011831, AB01-09-31, TSJ2004-065, IB19930612	Europe
	H2 (3)	TUB 011831, IB19850209, IB19930612	Europe
	H3 (6)	JFA 12428, IB19870221, TUB 011831, IB19850209, IB19930612, JFA 10065	Mountain (Colorado, Wyoming) Pacific (Washington)
	H4 (4)	JFA 10065	Pacific (Washington)
	H5 (1)	IB19890428	Pacific (Washington)
C. elegantior	H1 (11)	TUB 011388, TUB 011394, TUB 012709, IB19980414, IB20010192, IB19790599, AB00-09-120, AT2004126, AT2005138, IB20040204, IB19980248	Europe
	H2 (2)	IB19980414	Europe
	H3 (1)	IB20010192	Europe
	H4 (1)	AT2004277	Europe
	H5 (1)	JFA 12438	Mountain (Wyoming)
	H6 (13)	JFA 12438, IB19890189, IB19910141, IB19970249, IB19910140	Mountain (Wyoming)
	H7 (36)	IB19890226, IB19970300, IB19870057, IB19970107a, JFA 11452, IB19890059, IB19910140, IB1989226, IB19970249, IB19910140, JFA 11411	Mountain (Wyoming)
	H8 (8)	IB19970300	Mountain (Wyoming)
	H9 (4)	IB19970107a, JFA 11452, IB19890059, IB19910140, IB1989226	Mountain (Wyoming)

	H10 (4)	JFA 11693, TUB 019280, JFA 13226, JFA 13287	Pacific (Washington, Oregon)
	H11 (1)	JFA 13226	Pacific (Washington)
C. napus	H1 (23)	TUB 012717, S: F44393, TUB 019281, JFA 12426, IB19870275, IB19890298, IB19890479, IB19910237, IB19910261, IB19910270, IB19970162, IB10660, IB19970303, IB19870186, IB1997303b, IB19890242, TSJ2001-003, KH3, AT2005152, AT2005156	Europe Mountain (Wyoming, Colorado) Pacific (Washington, Oregon)
	H2 (3)	IB19870275, IB19890298	Mountain (Wyoming)
	H3 (1)	JFA 10070	Pacific (Washington)
