# Supplementary material for: Tracking the evolutionary history of Cortinarius species in section Calochroi, with transoceanic disjunct distributions
Source: BMC Evol Biol. 2011 Jul 19;11:213. doi: 10.1186/1471-2148-11-213 (PMC3161008; doi:10.1186/1471-2148-11-213)
Supplement: Additional File 2 — Population mutation rate, effective sample size (ESS), time of divergence and direction of migration estimates within C. arcuatorum, C. aureofulvus and C. elegantior population samples. The parameters are as follows: θ1, θ2 and θA, are the mean population rates for the Old World, the New World and the ancestral population, respectively; t is the mean time of divergence of populations from a common ancestor; m1 represents the mean number of migrations into the Old World and m2 is the mean number of migrations into the New World. Numbers in parenthesis are standard deviations. Reliable estimation of the highest probability density (HPD) intervals could not be achieved, as the posterior distribution turned out to be incomplete in several cases. All values represent arithmetic means taken from 15 independent runs per species. [file 1471-2148-11-213-S2.PDF]

Parameters	*C. arcuatorum*	*C. aureofulvus*	*C. elegantior*
$\boldsymbol{\theta_1}$	0.26 (± 0.04)	0.29 (± 0.00)	1.11 (± 0.01)
ESS	345	1328	3440
$\boldsymbol{\theta_2}$	4.29 (± 1.69)	2.12 (± 0.25)	1.45 (± 0.03)
ESS	61	1057	623
$\boldsymbol{\theta_A}$	52.23 (± 9.50)	50.78 (± 0.45)	7.57 (± 0.09)
ESS	568	19907	31361
**t**	0.51 (± 0.16)	5.43 (± 0.09)	5.36 (± 0.23)
ESS	151	3693	197
$\mathbf{m_1}$	3.58 (± 0.55)	3.06 (± 0.07)	0.34 (± 0.01)
ESS	251	4086	5030
$\mathbf{m_2}$	4.01 (± 0.21)	3.44 (± 0.10)	0.16 (± 0.00)
ESS	153	1766	1450
