# Supplementary material for: Tracking the evolutionary history of Cortinarius species in section Calochroi, with transoceanic disjunct distributions
Source: BMC Evol Biol. 2011 Jul 19;11:213. doi: 10.1186/1471-2148-11-213 (PMC3161008; doi:10.1186/1471-2148-11-213)
Supplement: Additional File 3 — Taxonomy. Macroscopical descriptions are based on fresh material, whereas microscopical structures were analysed from dried specimens. [file 1471-2148-11-213-S3.PDF]

Additional File 3

Title: Taxonomy

File format: PDF

Description of species: The morphological, macrochemical and ecological characteristics of the species presented in this paper are provided below. Based on our investigations the following species are recognized: 1) ***Cortinarius arcuatorum*** which occurs in Europe and in the Eastern Rocky Mountains (H4, H5); there are two new closely related species in the hardwood and mixed forests along the Pacific Coast, ***Cortinarius fulvo-arcuatorum*** California Northern populations (H3) and ***Cortinarius lilaciotinctus*** California (Mendocino) populations (H2) and a third new species occurs in Costa Rica associated with *Quercus,* ***Cortinarius jardinensis*** (H1); 2) ***Cortinarius aureofulvus***, which does not exhibit morphological divergence into additional species occurs disjunctly in Europe or western North America; 3) ***Cortinarius elegantior*** (H1, H2, H3, H4) occurs throughout much of Europe mainly in conifer forest; there two new species closely related species in western North America, ***Cortinarius elegantio-montanus*** Wyoming (H5, H6, H7, H8, H9) and ***Cortinarius elegantio-occidentalis*** Washington and Oregon (H10, H11), both also occur associated with conifers, especially *Picea*; and 4) ***Cortinarius napus*** which occurs disjunctly in parts of Europe and North America. This species is characterized by a wide variation in coloration of basidiomata in western North America (*C. albobrunnoides, C. albobrunnoides var. violaceovelatus* and *C. subpurpureophyllus var. sulphureovelatus* are synonyms of *C. napus*).

***Cortinarius arcuatorum* Rob. Henry, Bull. Soc. Mycol. Fr. 55: 80 (1939).**

Pileus 50 – 120 mm diam., convex or expanded, viscid, not typically innately fibrillose, but sometimes with single fibrils, sometimes with ochre velar plaques, especially at centre, soon orange brown to rose brown and with age becoming ocher reddish with dark brownish fibrils, margin often with lilac violet remains of the universal veil. *Context* whitish, or in bulb sometimes yellowish, sometimes violaceous in upper stipe. *Smell* none. *Taste* distinctive, slightly bitter. *Lamellae* adnexed, crowded, young greyish white to violaceous, in age grey brown or sordid rusty brown. *Stipe* 50 – 120 cm long, 9 – 25 mm thick, with a sharply marginate bulb up to 35 mm broad, bulb ocher, yellowish to yellow brownish, above often brownish violaceous especially toward the apex, later brownish. *Universal veil* at bulb margin violaceous, later in old specimens grey or grey brown.

Basidiospores 10 – 12 x 6 – 7 µm, Q = 1.54 – 1.83, mean = 1.70 and S = ± 0.10, amygdaliform to mainly citriniform, coarsely verrucose. *Basidia* 28 – 35 (–37) x 8 – 9 µm, clavate, 4-spored, slightly pinkish, sometimes with refringent contents. *Lamella trama* regular, composed of cylindrical hyphae 4 – 20 µm wide, pale pinkish. *Pileipellis* simplex. *Gelatinous layer of the pileus* well developed, sometimes with interwoven hyphae, composed of cylindrical hyphae 2 – 4 µm wide, colorless to pale pink. *Hyphae of epicutis* (3–) 4 – 5 (–6) µm wide, cylindrical, ascending into gelatinous layer, towards pileus trama more or less radially oriented, pink to slightly brown, hyphae sometimes faintly stripped with encrusting pigments. *Hyphae of pileus trama* cylindrical, in upper

strata pink, towards deeper strata yellowish pink with ovoid to subglobose elements.

Clamp connections present.

Chemical reaction: Fresh specimens: Pileus surface slightly rose or red brown with KOH. Context pink with KOH 30%. Universal veil at bulb margin pink, other parts of the bulb negative. White mycelium at base of bulb negative with KOH. Dried specimens: On pileus surface pink, sometimes becoming terracotta (IB19950564, IB19950596, IB19950686, IB19870239) and mycelia at the stipe base pink to intense wine red (IB19870107).

Specimens examined: Europe. **Germany.** Eschweiler (48.873684, 2.295048), *Fagus sylvatica*, September 14, 2008 TUB 019283 (GU363455), *Fagus sylvatica* and *Quercus* sp., October 03, 2001, TUB 011403 (AY174824); Tübingen, Großholz (48.873684, 2.295048), *Fagus sylvatica*, *Quercus* sp., and *Pinus sylvestris*, October 19, 2001, TUB 011421 (AY174822); Buir (48.873684, 2.295048), *Fagus sylvatica*, October 17, 1999, TUB 011447 (AY174823). North America. **USA.** Wyoming. Teton County. Teton National Forest, Trail to Arizona Lake (43.9613295, -110.619376), under *Picea engelmannii*, elevation 2200 m, August, 16, 1987, IB19870239 (GU363458).

Additional specimens examined: Europe. **Germany.** Mürlenbach (48.873684, 2.295048), growing associated with *Fagus sylvatica*, September 03, 2007, TUB 019278 (GU363456); Eschweiler, *Fagus sylvatica* and *Quercus* sp., September 30, 2007, TUB 019279 (GU363457). **Italy.** Prov. Parma, Ca'Bruna, Marzocco, near Borgotaro (48.873684, 2.295048), *Castanea* sp., *Quercus cerris*, October 07, 1996, IB19960216. North America. **USA.** Wyoming. Teton National Park. String Lake (43.786111, -

110.7325), under *Picea engelmannii* and *Pseudotsuga menziesii*, elevation 2090 m, July 19, 1987, IB19870107 (GU363459).

- Eastern Rocky Mountain population IB19870239 (GU363458): *Pileus* 45 – 80 mm diam., greyish blue, on marginal areas, more rarely up to disc, bluish grey (21B2), otherwise discoloured orange brown (59M-N), glutinous. *Lamellae* lilac, violet grey to greyish violet (17B2-3), than brownish lilac (29M), finally light milk coffee-brown, light grey to grey (near 7.5YR7/6). *Stipe* 30 – 70 mm long, 9 – 13 mm thick above, marginate bulb 18 – 27 mm thick, bluish grey, bluish white to bluish grey (20A2 to B2), bulb whitish ochraceous with velar remnants. *Veil* whitish ochraceous. *Context* whitish, bluish in cortex of apex, yellowish ochre in bulb. *Odor* none. *Taste* mild.

Basidiospores 10 – 12 x 6 – 7 μm, Q = 1.43 – 1.83, mean = 1.64 and S = ± 0.12, amygdaliform to ellipsoid, moderately verrucose. *Basidia* (32) 33 – 40 x 9 – 10 μm, hyaline, 4-spored. *Lamellae trama* regular, composed of cylindrical 3 – 20 μm hyaline hyphae. *Pileipellis* simplex. *Gelatinous layer of pileus* well developed, composed of cylindrical 2 – 4 μm, hyaline, wine red or pinkish hyphae. *Hyphae of the epicutis* 4 – 7 (–8) μm wide, cylindrical, hyaline to pale brownish. *Hyphae of the pileus trama* hyaline, yellow or pale brown.

Macrochemical reactions: Fresh specimens: KOH 30% red on pileus surface. Dried specimens: Gelatinous layer of the pileus in KOH 3% turning pink to wine red.

Cortinarius fulvo-arcuatorum **Garnica & Ammirati, sp. nov.** - California Northern populations (H3).

Pileo 38 – 70 mm lato, convexo, viscido, roseo-ochraceo dein rufulo-flavo ad rufulo-aurantiaco, lamellis adnexis, confertis ad aggregatis, purpurelleis ad pallide roseo-lilaceis, stipite 30 – 70 mm longo, apice 12 – 18 mm crasso, marginato-bulboso, pallide purpuro-lilaceae. Carne pilei albidae, stipitis albidae et lilacineae, odore fungoso, sapore amaro. Sporis (9) 10 – 12 x 6 – 7 µm, amygdaliformeis, verrucosis. In silvis Arbutus menziesii, Notholithocarpus densiflorus, Quercus chrysolepis, Pseudotsuga menziesii, Umbellularia californica, California, Del Norte County, Patrick Creek Campground, Holotypus JFA 11765 (WTU), 17 November 1995.

Pileus 38 – 70 mm diam., convex to plano-convex then uplifted, undulate, margin narrowly decurved to slightly upturned at edge, sterile tissue at edge persistently enrolled, glutinous, glabrous overall or margin with slight pale to faintly lilac veil remnants, not streaked, along edge tinted pink (7.5YR 7/4) to pinkish ochraceous but usually with some buff and faint lilac colors mixed in, otherwise reddish yellow (5YR6/6) to somewhat yellowish red (5YR 5/8) with some areas slightly paler colored, when mature somewhat brownish on disc and developing strong orange tones mixed with reddish yellow or dull reddish orange, in age reddish yellow orange or more orangish tones common. *Lamellae* deeply adnexed, arched then somewhat ventricose, up to 32 mm long, up to 6.5 mm wide, moderately thin, more or less crowded, easily split, edges more or less even, purplish to light pinkish lilac on faces and edges, developing slight brownish tints, gradually browner

but long retaining some lilac color. *Stipe* 30 – 70 mm long, 12 – 18 mm thick above, base 20 – 28 mm thick, bulbous, distinctly to strongly marginate, oblique to slightly flatten or somewhat depressed above rim, base rounded below and relatively short, surface above base silky fibrillose, pale purplish lilac (pale lilac) or white at apex and lilac below, basal mycelium white or slightly yellowish white, rim either pale purple to light purple lilac or somewhat brownish, veil fibrils and rim of bulb becoming coated rust brown from spores. *Context* to 9 mm thick on disc, gradually thinner to edge, solid and firm throughout, white, no lilac color in pileus, sometimes with lilac in stipe cortex near apex or in base, sometimes slightly discolored yellowish, brownish around larvae tunnels in stipe, where exposed by feeding snails strongly ochraceous. *Odor* fungoid. *Taste* bitter.

Basidiospores (9) 10 – 12 x 6 – 7 µm, Q = 1.57 – 1.93, mean = 1.70, S = ± 0.09 amygdaliform to broadly amygdaliform, moderately to coarsely verrucose, apex slightly extended. *Basidia* 32 – 38.5 x 8.0 – 10.5 µm, pinkish to hyaline or with concentrated reddish-pink pigment, clavate, 4-spored. *Lamella trama* regular, hyphae cylindrical to enlarged, 4 – 25 µm wide, hyaline to pinkish, some with reddish pigment. *Pileipellis* simplex. *Gelatinous layer of pileus* well developed, composed of interwoven, often ascending, 3 – 6.5 µm wide, cylindrical, hyaline to pinkish hyphae, often encrusted especially adjacent to epicutis. *Hyphae of epicutis* subparallel to interwoven, radially oriented, 4 – 10.5 µm wide, cylindrical but with some shorter, enlarged elements, pale yellowish buff to colorless, commonly encrusted; *Hyphae of pileus trama* 4.5 – 26 µm wide, cylindrical to enlarged, pink or slightly yellowish buff to hyaline, walls refractive. *Veil hyphae* 3 – 5 µm, cylindrical, slightly pinkish to hyaline. *Oleiferous hyphae* pinkish to greyish, common. *Clamp connections* present.

Macrochemical reaction: Fresh specimens: Reaction with 20% KOH red mixed with some brown on pileus surface, instantly pale red pink on pileus context, red on lamellae. Dried specimens: Pileus surface wine red, pink or terracotta and mycelia at the stipe base pink. In 3% KOH pileus and lamellae sections reddish pink.

Specimens examined: North America. **USA.** California. Del Norte County. Patrick Creek Campground (41° 52' 26.1", 123° 50' 40"), H.W. 199, gregarious, *Arbutus menziesii*, *Notholithocarpus densiflorus, Quercus chrysolepis, Pseudotsuga menziesii*, *Umbellularia californica,* November 17, 1995, JFA 11765 (Holotype), (EU057005), JFA 11766 (EU057004), and IB19950564 (GU363453); Big Flat Campground (41.68778, -123.90861), *Notholithocarpus densiflorus, Quercus* spp., *Pinus* sp., *Pseudotsuga menziesii*, November 21, 1995, JFA 11803 (EU057003) and IB19950596 (GU363454).

Cortinarius jardinensis **Garnica, Ammirati & Halling, sp. nov. -** Costa Rican populations (H1).

Pileo 44 – 80 mm lato, convexo vel lato-convexo, sicco ad viscido, bubalino vel roseo ad sublilacino dein brunneo ad rufulo-brunneo, lamellis adnexis, confertis ad aggregatis, violaceis ad purpuro-vinaceis, stipite 55 – 100 mm longo, apice 10 – 14 mm crasso, marginato-bulboso, albidae vel lilaceae. Carne pilei albidae, stipitis albidae et lilacineae, odore acerbo, sapore amaro. Sporis (11–) 12.5 – 14.5 (–15) x 6.5 – 7.5 µm, amygdaliformeis ad citriniformeis, verrucosis. In silvis Quercus copeyensis, Q. seemannii, Costa Rica, Prov. San José, Jardín de Dota, Holotypus JFA 12039 (USJ), 05 June 1996.

Pileus 44 – 80 mm diam., convex to plano-convex then plane to uplifted, more or less broadly subumbonate, becoming shallowly depressed, margin more or less decurved to applanate or somewhat uplifted to undulate in age, dry to moist or viscid, somewhat shiny, slightly innately fibrillose, disc and inner margin sometimes with small dry gluten patches, disc and inner margin brownish but with warm buff undertones at first, outer margin warm buff, dull yellowish buff or buff pink to faintly lilac, colors usually mixed with brownish, brownish Cinnamon Buff or slight Ochraceous Tawny tones, sometimes with brownish streaks, in age becoming more brownish with reddish brown areas (dark Mikado Brown) and orangish red brown tones. *Lamellae* adnexed, seceding in age, somewhat ventricose, close to crowded, to 40 mm long, to 9 mm wide, edges pale and even to irregular, moderately thick to thin, violet to purple vinaceous, becoming brownish violet, in age deep rich brown (rust brown mixed with slight Walnut Brown tones). *Stipe* 55 – 100 mm long, 10 – 14 mm thick above, base 14 – 23 mm thick, bulbous, marginate, more or less oblique or slightly depressed above distinct rim, rounded below, somewhat fragile, surface shiny white with lilac cast above bulb, sometimes upper half more lilac at first, in age stipe discoloring yellowish to brownish, with some superficial fibrils, universal veil on bulb rim white, basal mycelium and bulb whitish with ochraceous stains, in age stained darker ochraceous to yellowish or orangish. *Context* of pileus solid, firm, white to whitish, developing yellowish and brownish discolorations, to 13 mm thick in disc, gradually then abruptly thinner in outer margin, in stipe stuffed to hollow, white to whitish (lilac only in places at cortex surface as a line, no lilac in interior context), developing yellowish and brownish stains, becoming dark brown in age, especially around larva tunnels. *Odor* pungent. *Taste* bitter.

Basidiospores (11–) 12.5 – 14.5 (–15) x 6.5 – 7.5 µm, Q = 1.76 – 2.07, mean = 1.90, S ± 0.10, amygdaliform to citriniform, coarsely verrucose, with apex extended, smooth. *Basidia* 30 – 42 x 8 – 11 µm, pinkish to hyaline, clavate, 4-spored. *Lamella edges* with clavate to narrow cylindrical elements, mixed with basidia. *Lamella trama* regular, hyphae cylindrical to enlarged, 2.5 – 24 µm wide, hyaline to pinkish. *Pileipellis* simplex. *Gelatinous layer of pileus* well developed, composed of 2 – 7.5 µm wide, cylindrical, interwoven, hyaline to pale yellowish hyphae, but often containing yellow pigment, some spirally encrusted. *Hyphae of epicutis* 4 – 12.5 µm wide, cylindrical to somewhat enlarged, interwoven in upper portion, in lower portion more radially arranged, yellowish buff to colorless, some filled with yellow orange brown granules, frequently spirally encrusted, especially in upper layer, septa walls often yellowish thickened. *Hyphae of pileus trama* 3 – 25 µm wide, cylindrical to enlarged, pinkish to hyaline, walls strongly refractive. *Oleiferous hyphae* cylindrical to irregular in outline, pinkish, abundant in pileus trama. *Veil hyphae* 2.5 – 5.5 µm wide, cylindrical, pinkish to hyaline, or occasionally yellowish. *Clamp connections* present.

Macrochemical reaction: Fresh specimens: Reactions with 20% KOH watery red to bright pink-red on pileus surface, bright red on context of pileus and stipe base. Dried specimens: Pileus surface wine red and mycelia at the stipe base pinkish. With 3% KOH section of pileus and lamellae of dried specimens reddish pink.

Specimens examined: Central America. **Costa Rica.** Prov. San José, Jardín de Dota, 3.5 km west of InterAmerican Highway at Empalme (9° 42' 52" N, 83° 58' 28" W), scattered to gregarious, *Quercus copeyensis*, *Q. seemannii*, 2220 m, June 05, 1996, collectors J. Torres, R. Halling, J. Ammirati, JFA 11918 (USJ), JFA 11919; same location, June 13,

1996, collectors R. Halling, J. Ammirati, JFA 12037 (USJ) (EU057060), JFA 12038 (USF), JFA 12039 (Holotype, USJ) (EU057000); same location, June 05, 2004, collectors R. Halling, M. Neves, C. Ovrebo, J. Ammirati, JFA 12966 (INB), JFA 12972 (INB). Prov. Cartago, Parque Prusia (9° 57' 56" N, 83° 52' 15" W), gregarious, *Quercus costaricensis,* 2900 m, June 16, 1996, collectors J. Ammirati, R. Halling, JFA 12061 (USJ) (EU057001).

***Cortinarius lilaciotinctus* Garnica & Ammirati, sp. nov. -** California (Mendocino) populations (H2).

Pileo 36 – 42 mm lato, convexo, viscido, rufulo-aurantiaco-brunneo ad brunneo-roseo cinnamomeo, lamellis adnexis, confertis ad aggregatis, pallide griseo-brunneis vel sublilaceis, stipite 43 – 52 mm longo, apice 9 – 11 mm crasso, marginate-bulboso, pallido violaceae, lilaceo-tincto. Carne pilei albidae, stipitis albidae et lilacineae, odore nullo, sapore amaro. Sporis 10 – 11 x 5 – 6.5 µm, amygdaliformeis ad subcitriniformeis, verrucosis. In silvis Notholithocarpus densiflorus, Pseudotsuga menziesii, Tsuga heterophylla, Sequoia sempervirens, California, Mendocino County. Jackson State Forest, Mendocino, Holotypus IB19950686 (IB), 08 December 1995.

Pileus 36 – 42 mm diam., hemispheric to convex, margin incurved to decurved, edge enrolled, viscid to glutinous, with a thin whitish coating on portions of margin and disc, reddish orange brown (Mikado Brown) to orange cinnamon, brownish vinaceous cinnamon or brownish pinkish cinnamon (Pinkish Cinnamon, Onion Skin Pink,

Vinaceous Tawny to near Pecan Brown), at edge very pale pinkish tan, generally becoming paler in age. *Lamellae* adnexed, up to 16 mm long, up to 6.5 mm wide, not ventricose, moderately thin, close to crowded, pale grey to brownish or watery grey brown, sometimes with a lilac white tint, edges lilac to whitish to light brown, uneven. *Stipe* 43 – 52 mm long, 9 – 11 mm thick above, base 20 – 25 mm wide, bulbous, marginate, rounded to tapered below, above bulb Pale Mauve to Pale Lavender Violet, darkening to rich lilac where handled, darker where handled on rim of bulb, basal mycelium bluish lavender to lilac at first, in age base whitish to brownish discolored, veil bluish lavender to lilac. *Context* to 8 mm on disc, gradually thinner to edge, white throughout except for lilac along cortex of stipe, becoming brownish to watery grey brown in age. *Odor* none. *Taste* bitter.

Basidiospores 10 – 11 x 5 – 6.5 µm, Q = 1.54 – 2.20, mean = 1.76 and S = ± 0.12, amygdaliform to subcitriniform or sometimes citriniform, moderately verrucose. Basidia 25 – 33 x 7 – 9 µm, hyaline to pale pinkish, 4-spored. Lamella trama regular, hyphae 3 – 16 µm wide, cylindrical to broadly cylindrical, yellow with pinkish tinge. Pileipellis simplex. Gelatinous layer of pileus well developed, composed of 2 – 4 µm wide, cylindrical, pale yellow to pinkish hyphae. Hyphae of epicutis 4 – 6 µm wide, cylindrical, hyaline, pinkish or brownish. Hyphae of pileus trama (3–) 4 – 15 µm wide, upper strata pink, deeper strata yellow pinkish. Oleiferous hyphae present. Veil hyphae 2.5 – 6 µm wide, cylindrical, colorless. Clamp connections present.

Macrochemical reaction: Fresh specimens: Reaction with 20% KOH red on pileus surface and lamellae. Dried specimens: Pileus surface wine red to pink terracotta and

mycelia at the stipe base pink. With 3% KOH sections of lamellae and pileus surface (especially the gelatinous layer) pink.

Specimens examined: North America. **USA.** California. Mendocino County. Jackson State Forest, Mendocino (39.307778, -123.799444) near intersection of Roads 408 and 409, gregarious, *Notholithocarpus densiflorus*, *Pseudotsuga menziesii*, *Tsuga heterophylla*, and *Sequoia sempervirens*, December 08, 1995, JFA 11893 (Isotype) (EU057002) (= IB19950686 Holotype), (GU363452).

Comments: Above are descriptions of *Cortinarius arcuatorum* and three new species, *C. jardinensis*, *C. lilaciotinctus* and *C. fulvo-arcuatorum* in the Dibaphi clade, Section *Calochroi, that* span a broad geographical range from Europe to western North America and Costa Rica. In general these species tend to be strongly associated with members of the Fagaceae, except for the populations of *C. arcuatorum* from the eastern Rocky Mountains, where only conifers occur. European populations of *C. arcuatorum* come from forest of *Fagus, Fagus* and *Quercus* or *Castanea* and *Quercus* although in some forests *Pinus* may be mixed in with *Fagus* and *Quercus*, typically on calcareous soils between the 200 to 400 m of altitude. According to Moser [1], *C. arcuatorum* may also occur in association with Betulaceae (*Carpinus*). In Europe, *C. arcuatorum* is somewhat frequent and widely distributed being collected from various sites in Denmark, France, Germany, Italy, Sweden and Switzerland. *Cortinarius fulvo-arcuatorum* and *C. lilaciotinctus* occur in mixed forests. The former occurs in forests with *Tsuga*, *Pseudotsuga* and *Notholithocarpus*, and the latter in forests with *Arbutus, Notholithocarpus, Quercus* and *Pseudotsuga* or with *Notholithocarpus, Quercus, Pinus* and *Pseudotsuga.* In the Eastern Rocky Mountains *C. arcuatorum* occur in conifer forests

either with *Picea engelmannii* or with *P. engelmannii* and *Pseudotsuga. Pinus* and *Abies* also may be present in these forests as well. Therefore, a strict association of these populations with *Picea* cannot be fully confirmed with the limited material analysed to date. *Cortinarius jardinensis* is associated with *Quercus*, including *Q. costaricensis* and *Q. seemannii* (section *Lobatae*) and *Q. copeyensis* (section *Quercus*).

Morphologically, the pilei of European *C. arcuatorum*, *C. fulvo-arcuatorum* and C. *lilaciotinctus* have pink ocher to ocher red colors mixed with pinkish brown and orange brown to reddish orange brown colors. In *C. jardinensis* the pileus margin is more yellowish buff sometimes with slight lilac tones on the edge, and the center is more brown to reddish brown. The Eastern Rocky Mountain populations of *C. arcuatorum* have greyish blue pilei that discolor orange brown giving the appearance of a species in the *C. caesiocanescens* group. Violet, purple and lilac colors are variable for the lamellae across all population samples, but most populations express at least some lilac tones with the strongest blue to violaceous colors in the Eastern Rocky Mountain populations of *C. arcuatorum*, Costa Rica and *C. fulvo-arcuatorum*. Blue to violet colors are common on the stipe of all populations, and in *C. lilaciotinctus* this color intensifies to dark violet following handling. Veil color varies from white to ocher or pale lilac but is more strongly lilac to blue lavender in California populations (H2, H3).

Cortinarius arcuatorum typically has a bitter taste, except for the eastern Rocky Mountain population, which has a mild taste. All species in the Dibaphi clade give a red to pink reactions of the basidiomata with KOH. *Cortinarius arcuatorum* is closely related to *C. dibaphus* sharing a very similar stature and general coloration, taste and KOH

reactions, but differs by the lacks of violet to lilac colors on the pileus. Ecologically, *C. arcuatorum* primarily occurs in broadleaf forests, while *C. dibaphus* is restricted to coniferous trees, specifically with *Abies alba*. As part of this study, we sequenced several collections from IB herbarium identified as *C. dibaphus* collected from broadleaf forests that rather correspond to other separate species.

Genetic distance comparisons based on 621 bp of ITS region show that Costa Rican populations (H1) are the more closely related to *C. dibaphus* (4.7% divergence), whereas from some California populations (IB19950564) (H3) are less closely related to it (7.3% divergence), and the European and Wyoming (IB19870107, IB19870239) (H4 + H5) populations (7.2%), respectively.

In general, species in the Dibaphi clade described here show relatively low morphological divergence from one another making it difficult to recognize species using only traditional taxonomic features. However, the geographical distributions of these species are yet not completely known, and therefore, there may be some additional distinctive morphological features that permit the recognition of these populations and species in the field. Because patterns of coloration of the basidiomata from European populations of *C. arcuatorum* are somewhat similar in appearance to *C. lilaciotinctus*, both *C. lilaciotinctus* and *C. fulvo-arcuatorum* were included under *C. arcuatorum* [2]. European populations of *C. arcuatorum* are rather variable in stature and coloration of the basidiomata. The taxon *C. fulvoincarnatus* Joachim characterized by slender and vivid colored basidiomata was found to be co-specific with *C. arcuatorum* based on ITS

sequences analyses [3]. Based on the known populations to date only the two North American species in California are close enough to one another to be considered sympatric.

Microscopically, basidiospore size of all species is rather uniform, generally 10 – 12 x 6 – 7 µm, the exception being *C. jardinensis* which has significantly large spores, 12 – 15 x 6.5 – 7.5 µm. Across all species basidiospore shape varies from amygdaliform to citriniform and the ornamentation is typically rather strongly verrucose.

Cortinarius aureofulvus **M. M. Moser, Sydowia 6: 154 (1952).**

Pileus 40 – 100 mm diam., convex to plano-convex, broadly umbonate, margin incurved to decurved, viscid, not innately fibrillose, but often stripped by formerly adhering coniferous leaves, often with brownish velar plaques, especially at centre, young or later at margin pale yellow, yellow or greenish yellow, at centre ochraceous yellow, with age soon orange to orange brown, brownish orange or reddish brown. *Context* especially in upper stipe cortex and in bulb yellowish, otherwise young white, pale blue grey, pale violaceous or grayish. *Odor* none, fungoid or slightly fragrant, tatse mild to fungoid. *Lamellae* adnexed, crowded, young yellow, lemon yellow or greenish yellow or yellow with a grey tinge, in age rusty brown. *Stipe* 30 – 90 mm long, 7.5 – 18 mm thick, with a marginate bulb of up to 30 mm broad, at apex greenish yellow, otherwise yellowish to yellow, rim of bulb becoming orange brown to orange red brown. *Basal Mycelium* whitish to yellowish becoming brownish. *Universal veil* at bulb margin greenish yellow,

later orange brown. *Basidiospores* (9–) 10 – 13 x (5.5–) 6 – 7 (–7.5) µm, Q = 1.45 – 1.91, mean = 1.69 and S = ± 0.09, amygdaliform to mostly citriniform, moderately to coarsely verrucose. *Basidia* 33 – 48 x 8 – 12 µm, clavate, 4-spored, hyaline to slightly wine red, older basidia with wine red content. *Lamella trama* regular composed of hyphae cylindrical, 2.5 – 17.5 µm wide, wine red. *Pileipellis* simplex. *Gelatinous layer of pileus* well developed, composed of 2 – 5.5 µm wide, hyaline to wine red - purple hyphae. *Hyphae of epicutis* 4 – 14 µm wide, cylindrical, brownish to wine red. *Hyphae of pileus trama* 5.5 – 30 µm wide, cylindrical, slightly wine red in upper strata, yellow in lower strata. *Oleiferous hyphae* greyish, scattered. *Veil hyphae* 2.5 – 4.5 µm wide, cylindrical, pinkish to hyaline, some slightly encrusted. *Clamp connections* present.

Macrochemical reactions: Fresh specimens: Pileus surface dark reddish to vinaceous brown; on yellow parts of context reddish to blood red.; on basal mycelium light vinaceous. Dried specimens: Black on both pileus surface and basal mycelium.

Specimens examined: Europe. **Germany.** Wolterdingen (53°1'53"N 9°49'58"E), growing associated with *Picea abies*, October 11, 1991, TUB 011831 (AY669571). North America. **USA.** Colorado. Boulder County. Mountain Research Station, Como Creek, gregarious under *Picea engelmannii, Pinus contorta, Populus tremuloides*, and *Salix* sp., August 18, 1997, JFA 12428 (EU0567061). Washington. Skagit County. Easy Pass Trailhead (48.5876, -120.8021), *Abies lasiocarpa*, *Picea engelmannii*, September 06, 1989, IB19890428 (GU363497); *loc. cit.*, scattered to gregarious in deep litter, mixed forest of *Abies lasiocarpa*, *Picea engelmannii*, *Pinus monticola*, *Pseudotsuga menziesii,* and *Tsuga mertensiana*, September 12, 1989, JFA 10065 (EU0567051) and *Abies lasiocarpa, Pseudotsuga menziesii* IB19890474.

Additional specimens examined: Europe. **Austria.** Kärnten, near Knappenberg (48.873684, 2.295048), *Picea* sp. forest, September 30, 1993, IB19930612 (GU363495). **Sweden.** Dalarna (48.873684, 2.295048), Rättivik, *Pinus* sp. forest on calcareous soil, August 14, 1985 IB19850209 (GU363494). North America. **USA.** Wyoming. Teton National Forest, Turpin Meadow, close to the Creek, *Picea* sp., August 13, 1987, IB19870221 (GU363496). Flagstaff Rd. "Calypso Creek", Teton National Forest, *Picea engelmannii*, September 01, 1991, IB19910396.

Comments: Populations of *Cortinarius aureofulvus* occurring disjunctly in regions of Europe and North America are morphologically readily recognizable in the field and microscopically they are essentially the same [4]. Populations of this taxon are rather infrequent in North America and Europe and are rather restricted to subalpine sites associated with coniferous trees. In western North America, populations have been found in conifer forests in the Olympic, Cascade and Rocky Mountains. Therefore, based on current field records, its natural range of distribution in North America includes forests in subalpine areas of Washington (Pacific), Wyoming and Colorado (eastern Rocky Mountains). The Wyoming populations are in forests with *Picea,* often mixed with *Abies* and *Pinus*; in Washington they occur in mixed conifer forests of *Picea* and *Abies, Abies* and *Pseudotsuga,* or *Abies, Picea, Pinus, Pseudotsuga* and *Tsuga*; in Colorado they occur with *Picea* and *Pinus* mixed with *Salix* and *Populus*; and in Europe they are associated with *Picea* and *Pinus*.

Phylogenetically, *C. aureofulvus* represents an isolated line of speciation within the section *Calochroi* (data not shown).

Cortinarius elegantior **(Fr.) Fr., Epicr. Syst. Mycol. (Upsaliae), (1838).**

Pileus 60 – 150 mm diam., convex, young yellow, yellow brown or orange brown, later mostly olivaceous brown, with age darker brown, intensely innately brownish fibrillose, often with pale yellow, pale grey, grey brown or brown velar plaques, especially at centre, not hygrophanous, viscid. *Lamellae* adnexed, margin slightly serrate, young straw yellow, greenish yellow or yellow, in age brown. *Stipe* 60 – 120 mm long, 16 – 30 mm thick, with a marginate bulb of up to 50 mm broad, pale yellow to yellow, later brown. *Universal veil* at the bulb margin yellowish to ochre yellow, later brown. *Context* pale yellow, but in lower part of the stipe and in bulb often darker yellow or orange-yellow. *Odor* none.

Basidiospores 13 – 15 x 7.0 – 9.0 µm, Q = 1.63 – 2.00, mean = 1.81 and S = ± 0.10, citriniform, very coarsely verrucose. *Basidia* 35 – 45 x 10 – 12 µm, clavate, 4-spored, hyaline to slightly wine red, older basidia with wine red contents. *Lamella trama* regular composed of hyphae cylindrical, 4 – 15 µm wide, slightly wine red. *Pileipellis* simplex. G*elatinous layer of pileus* composed of 3 – 5 wide, pale yellow, well developed. *Hyphae of epicutis* 5 – 8 µm wide, cylindrical, yellow brown to with wine red tinge, sometimes faintly stripped, well developed. *Hyphae of pileus trama* composed of cylindrical to enlarged, upper strata slightly reddish brown and deeper strata yellow. *Hyphae of veil* 2 – 3 µm wide, collapsed, yellowish. *Clamp connections* present.

Macrochemical reaction: Fresh specimens: Pileus surface red brown and context of the bulb in young specimens reddish to wine red (with 25% ammonia intensely red), otherwise in stipe and pileus negative, brownish or weakly reddish. Veil at base of the

bulb whitish, yellowish or greenish, slowly red. Dried specimens: Pileus surface wine red to red brown and mycelium at the stipe base wine red.

Specimens examined: Europe. **Austria.** Tannheim (48.873684, 2.295048). *Picea abies*, October 03, 2003 TUB 012709 (EF014262). **Germany.** Oberjoch (48.873684, 2.295048), *Picea abies*, October 2000, TUB 011388 (AY174850) and TUB 011394 (AY174851).

Additional specimens examined: Europe. **Austria.** Tirol, Arztal (48.873684, 2.295048). *Picea abies*, September 29, 2004, IB20040204 (GU363463); near Halltales, *Picea abies*, September 29, 2004, IB19790599 (GU363462). **Germany.** Waldhausen (48.873684, 2.295048), Kimbergsee, *Pinus sylvestris*, *Picea abies* and *Abies alba*, October 09, 2005 TUB 019300. **Italy.** Val di Sella (48.873684, 2.295048), Trentino, *Abies* sp., *Fagus* sp., *Picea* sp., September 23, 1993, IB19930230; Monte Rvero, *Picea* in open meadow forest, September 09, 1996, IB1996/0097. **Norway.** Oppland, Lunner (48.873684, 2.295048), *Picea abies* on calcareous soil, August 22, 1977, IB19770272. **Switzerland.** Kanton Solothum, Matzendorf (48.873684, 2.295048), *Picea* sp., *Pinus* sp., September 27, 2001, IB20010192 (GU363461); Kanton Bern, road between Axalpe (48.873684, 2.295048) and Hinterburgseeli, *Abies alba*, *Fagus sylvatica*, *Picea abies*, September 25, 1977, IB19770113; VD, Forêt de Berley, Montagny-les Monts (48.873684, 2.295048) with *Abies alba*, *Fagus sylvatica*, September 30, 1998, IB19980248 (GU363464); Kanton Graubünden, Valbella (48.873684, 2.295048), 1500 m. *Picea* sp., September 17, 1998, IB19980414 (GU363460); Kanton Solothurn, Matzendorf, above Längenmoos (48.873684, 2.295048), *Picea* sp., *Pinus* sp., September 27, 2001, IB20010192 (GU363461). The above material represents the European populations (H1, H2, H3, H4).

Cortinarius elegantio-montanus **Garnica & Ammirati sp. nov., stat. nov., -** Eastern Rocky Mountain population (H5, H6, H7, H8, H9).

Synonym:

Cortinarius elegantior Fr. var. *americanus* M. M. Moser & McKnight, Mycotaxon **55**:318–321 (1995).

Pileo 43–120 mm lato, convexo ad plano-convexo, margine luteo ad ochraceo, discum brunneo, aurantio-brunneo ad rufulo-aurantio-brunneo, lamellis adnexis, aggregatis, olivaceo-luteis ad sordido-pallide luteis, stipite 37–70 mm longo, apice 18–30 mm crasso, marginate-bulboso, pallide luteae. Carne pilei pallide lutea, stipitis, lutea, in basi transeuns rufulo-ochracea, aurantiaca ad roseo-aurantiaca, odore acerbo vel nullo, sapore miti. Sporis (10–) 13 – 15 (–15.5) x (6.5–) 8 – 9.5 (–10.5) µm, amygdaliformeis ad citriniformeis, crasse verrucosis. In silvis Picea pungens, *Picea engelmannii*, *Wyoming, Teton County, Turpin Meadow, Holotypus IB19890059 (IB), July 17, 1989.*

Pileus 43 – 120 diam., hemispheric then convex to convex flattened, edge enrolled, sticky to dry, outer margin often somewhat streaked with darker brown colors, some areas of inner margin and disc breaking into small patches, edge and outer margin pale yellow to light yellow to ochraceous or duller warm buff, inward brownish or dull orange brown to red orange brown, often a light crust brown at center, developing strong brownish to foxy colors on center. *Lamellae* adnexed, narrow, up to 6 mm wide, crowded (close to crowded), yellow with slight olivaceous hue or light rich ochraceous at first (a dull light

yellow), gradually more brownish (almost an olive tone to brown color), often with rusty spots. *Stipe* 37 – 70 mm long, 18 – 30 mm thick above, base bulbous, 25 – 42 mm thick, with rounded margin, not flattened or depressed on rim, often tapered below to a point, basal mycelium white but with ochraceous areas, surface above base pale yellow but developing ochraceous to dark yellow or watery yellow streaks below, on bulb edge becoming yellowish to somewhat brownish or with slight orangish or pinkish colors mixed with brown. *Veil* pale yellow (inner veil whitish, pale), sulfur yellow to strongly yellow ochraceous matted fibrillose on bulb margin. *Context* up to 15 mm thick disc, thin in outer margin (edge), pale yellowish or yellowish white or ochraceous white throughout with some watery or darker yellow areas in stipe cortex or apex, after 5 – 10 minutes of exposure developing reddish ochraceous, orange or pinkish orange colors, becoming brownish around larvae tunnels, and with age brownish in base of stipe. *Odor* pungent or not distinctive. *Taste* of pileus context mild.

Basidiospores (10–) 13 – 15 (–15.5) x (6.5–) 8 – 9.5 (–10.5) µm, Q = 1.3 – 1.8, mean = 1.58, S = ± 0.09, amygdaliform to mostly citriniform, coarsely to very coarsely ornamented, apex extended, snout-like, smooth. *Basidia* 30 – 56 x 9 – 13 µm, hyaline or containing reddish granules, clavate, 4-spored. *Lamellar edges* occasionally with irregular, clavate to subcapitate elements. *Lamella trama* regular, hyphae 2.5 – 25 µm wide, cylindrical to somewhat enlarged, colorless, often with hyaline granules, some containing reddish to orange reddish granules. *Pileipellis* simplex. *Gelatinous layer of pileus* composed of interwoven, 2 – 9 µm wide, well developed, hyaline to slightly yellowish, often encrusted hyphae. *Hyphae of epicutis* more or less radially arranged, interwoven, 3 – 16 µm wide, cylindrical to somewhat enlarged, hyaline to yellowish or

pinkish to pinkish ochraceous, often spirally encrusted, rarely containing yellow brown pigment. *Hyphae of pileus trama* 4.5 – 25 µm, cylindrical to enlarged, hyaline, rarely encrusted, rarely containing yellow brown pigment. *Oleiferous hyphae* greyish or pinkish, fairly common. *Veil hyphae* 2.5 – 6 µm wide, cylindrical, hyaline, few faintly yellowish. *Clamp connections* present.

Macrochemical reactions: Fresh specimens: With 30% KOH on flesh in stipe base deep red then dull wine red, pinkish on pileus flesh, light reddish on pileus surface, dull pinkish on lamellae. Vapor of NH_4OH pink in flesh of bulb. Dried specimens: wine red on pileus surface, and basal mycelium.

Specimens examined: North America. **USA.** Wyoming. Albany County. Snowy Range, intersection of HW 287 and Road 221, below Silver Lake (41.31278, -106.35889), gregarious, *Picea engelmannii, Pinus contorta*, August 19, 1997, JFA 12438 (EU056999). Fremont County. Falls Campground, Brooks Lake area, *Picea engelmannii* and *Pinus contorta*, gregarious in conifer duff, August 12, 1989, JFA 9934; Brooks Lake (43.751133, -110.003743), first creek beyond ranch, gregarious to caespitose in deep humus, *Abies lasiocarpa*, *Picea engelmannii, P. pungens, Pinus contorta,* August 27, 1995, JFA 11411 (GU363471). Teton County. Turpin Meadow (43.855, -110.26222), *Picea pungens*, *P. engelmannii*, July 17, 1989 IB19890059 (Holotype) (GU363473); Fourmile Meadow, creek behind beaver pond, *Picea engelmannii,* August 7, 1989, IB19890189 (GU363468); above Turpin Meadow Lodge, *Picea engelmannii, P. pungens*, July 29, 1991, IB19910141 (GU363470); Flagstaff Road, 2.5 miles from HW, dry conditions, gregarious, buried in humus layer *Picea engelmannii, Pinus contorta*, September 01, 1995, JFA 11452 (EU056998).

Additional specimens examined: North America. **USA.** Wyoming. Teton County. above Turpin Meadow, *Picea engemannii, P. pungens*, July 23, 1987, IB19870057 (GU363467) and July 29, 1991, IB19910140 (GU363474); Two Ocean Mountain, *Picea engelmannii*, August 31, 1997, IB1997030 (GU363466); Fourmile Meadow (43.8172, -110.2607), *Picea engelmannii, Abies lasiocarpa,* August 02, 1997, IB19970107a (GU363472); Flagstaff Rd., about 1 mile from eastern end, *Picea engelmannii*, August 12, 1989, IB1989226 (GU363475); Teton National Park (43.733333, -110.803333). Granite Creek Middle Fork above Patrol Cabin, *Picea engelmannii*, *Abies lasiocarpa*, August 25, 1997, IB19970249 (GU363469).

***Cortinarius elegantio-occidentalis* Garnica & Ammirati, sp. nov.** - Western (Pacific) populations (H10, H11).

Pileo 65 – 150 mm lato, hemispherico vel lato-convexo, visido ad sicco, margine pallide luteo ad sordide ochraceo-aurantio-luteo, discum sordide pallide luteo ad luteo-brunneo, aurantio-brunneo et rufulo-brunneo, lamellis adnexis, aggregatis, pallide luteo ad brunneo-olivaceo-luteis, stipite 55 – 80 mm longo, apice 20 – 30 mm casso, marginate-bulboso, pallide luteae. Carne pilei pallide lutea ad lutea, stipitis lutea, in basi transeuns pallide rosea an inmutabile, odore acerbo vel mitis, sapore mitis. Sporis (12–) 12.5 – 15.5 (–16.5 – 18) x (7–) 7.5 – 9 (–10) µm, citriniformeis ad amygdaliformeis, crasse verrucosis. In silvis Abies lasiocarpa, Larix occidentalis, Picea engelmannii, Pinus contorta, Pseudotsuga menziesii, Washington, Kittitas County, Table Mountain, Holotypus JFA 13226 (WTU), 31 August, 2008.

Pileus 65 – 150 mm diam., broadly hemispheric to broadly convex, becoming convex with flattened disc or sometimes shallowly depressed, margin decurved with strongly enrolled edge, becoming uplifted undulate in age, viscid at first, then dry, margin shiny, edge distinctly pale yellow to light yellow or dull ochraceous orange yellow, margin pale yellow to ochraceous, in places tinted brownish, with numerous small innate streaks, disc dull light yellowish buff (pale Isabella Color) with some small brownish patches, sometimes with patches of reddish brown gluten, in age developing olivaceous to dull yellow brown, orange brown and reddish-brown colors overall, margin sometimes developing dark reddish brown streaks. *Lamellae* adnexed, crowded, becoming broadest near base, pale yellow becoming brownish olive yellow, edges uneven and pale- to light yellow. *Stipe* 55 – 80 mm long, 20 – 30 mm thick above, base 35 – 55 mm thick, bulbous, marginate, silky fibrillose, pale yellow to yellow white, some surface fibrils more yellow, basal mycelium pale yellow to whitish or white or some areas more ochraceous, bulb margin whitish, light yellow to ochraceous or rusty ochraceous, sometimes veil leaving pale yellow remnants or viscid yellowish membrane on bulb rim. *Partial veil* pale colored. *Context* solid, firm, yellow to light yellow or yellow white, sometimes with watery yellow or orange yellow along cut stipe cortex, yellow color becoming darker and duller after long exposure, in base with white line at soil, interior of stipe base typically does not become pink on exposure (10 – 15 minutes after cutting), but after many hours of exposure sometimes becoming slightly pinkish. *Odor* pungent to slight. *Taste* pleasant to mild.

Basidiospores (12–) 12.5 – 15.5 (–16.5 – 18) x (7–) 7.5 – 9 (–10) µm, Q = 1.47 – 2.0, mean = 1.68, S = ± 0.11, citriniform or less commonly amygdaliform, coarsely verrucose,

with apex typically strongly extended, smooth, sometimes curved. *Basidia* 40 – 44 x 9.5 – 14.5 µm, hyaline or with yellow to yellowish brown or reddish granules, clavate, 4-spored. *Lamella trama* regular, hyphae cylindrical to enlarged, 4 – 24 µm wide, hyaline or with yellowish orange brown pigment, sometimes encrusted or with yellowish walls. *Pileipellis* simplex. *Gelatinous layer of pileus* well developed, composed of up to 4 – 10 µm wide, cylindrical hyphae, hyaline or slightly yellow, some containing orange brown pigment, often spirally encrusted. *Hyphae of epicutis* radially oriented, interwoven, 3 – 13 µm wide, cylindrical to broadly cylindrical, hyaline to yellowish, walls colorless to yellowish, spirally encrusted, some containing orange brown pigment, with yellow pigment in between hyphae. *Hyphae of pileus trama* cylindrical to enlarged, 4.5 – 34.5 µm wide, colorless, rarely yellowish or spirally encrusted. *Oleiferous hyphae* scattered, yellowish. *Veil hyphae* 4 – 6.5 µm, cylindrical, hyaline to yellowish, walls refractive, some encrusted. *Clamp connections* present.

Macrochemical reaction: Fresh specimens: Reactions (somewhat variable in intensity) pinkish to reddish or watery red on pileus surface; reddish to pinkish in context of pileus and stipe, but reddish reaction sometimes slight in whitish yellow areas of pileus context; pinkish to reddish on bulb and lower stipe surface. Dried specimens: Pileus surface wine red to dark red brown and mycelia at the stipe base wine red or pale pink (JFA 11693). With 3% KOH sections of lamellae slightly pinkish. Collection JFA 11693 NH_4OH conc. and 20% KOH – both pinkish in stipe base, pileus surface dark brown and context pinkish.

Specimens examined: North America. **USA.** Washington. Kittitas County. Table Mountain (47.252624, -120.585353), *Abies lasiocarpa, Larix occidentalis, Picea*

engelmannii, Pinus contorta, Pseudotsuga menziesii, August 31, 2008, JFA 13226 (Holotype) (GU363477), JFA 13227, JFA 13228, JFA 13229, JFA 13231; Naneum Meadow (47.2907, -120.5467), gregarious, *Abies lasiocarpa, Picea engelmannii, Pinus contorta, Pseudotsuga menziesii*, September 09, 2008, JFA 13239; loc. cit., *Abies lasiocarpa, Picea engelmannii, Pinus contorta*, September 11, 2008, JFA 13287 (GU363478).

Additional specimens examined: North America. **USA.** Oregon. Lincoln County. Drift Creek Organization Camp (44.4272, -123.98211), solitary, *Picea sitchensis, Tsuga heterophylla*, November 06, 1995, JFA 11693 (EU056997). Washington. Snohomish County. Barclay Lake Trail (47.7854, -121.4279), gregarious, mixture of *Abies amabilis, Pseudotsuga menziesii*, *Tsuga heterophylla,* October 11, 2006, *leg.* S. Garnica & J. Ammirati, TUB 019280 (GU363476).

Comments: The *Cortinarius elegantior* lineage is represented here by three species namely *C. elegantior*, *C. elegantio-montanus* and *C. elegantio-occidentalis* that are morphologically very similar. All these species are characterized by their yellow brown to brownish yellow or orange brown to brownish pileus, yellow lamellae, stipe and veil, yellowish white to yellow or orange yellow context, flesh in the stipe base that becomes pinkish on exposure, and parts of the basidiomata that turn red to vinaceous with the application of KOH. The basidiospores are distinctly citriniform, large (13 – 15 x 7 – 9 μm) and coarsely ornamented. It is closely related to the European *C. parafulmineus* R. Henry, which is associated with *Pinus sylvestris* in subalpine sites. Genetic distance comparisons based on 638 bp of ITS region support a closely relationship between the Wyoming population JFA 11411 of *C. elegantio-montanus* and *C. parafulmineus* (3.0%

divergence), whereas the populations IB19910141, IB19970300, IB19890189 and JFA 12438 from Wyoming and the European population UDB000721 of *C. elegantior* appeared to be less related with a divergence of 3.8%.

Cortinarius elegantior has been reported from both conifer (*Picea, Pinus, Abies*), mixed and broadleaf (*Fagus*, *Quercus*, *Betula*, *Corylus*) forests in Europe [5], but it seems to be primarily associated with *Picea*. The name *C. elegantior* has been applied to western North American populations associated with conifers, including *Picea*, where the elegantior clade is especially common in subalpine forests. *Cortinarius elegantior* var. *americanus* was recognized as a member of the Rocky Mountain mycota, differing from var. *elegantior* by sulphur yellow veil, paler yellow pileus lacking olivaceous tones, and the color of the context which is yellowish but not with deep rhubarb color in the base of the stipe. Also, another noted difference was the lack of small veil patches on the pileus surface which is characteristic of European material.

The allied species to *Cortinarius elegantior* described here are somewhat difficult to separate from one another morphologically and ecologically. *C. elegantio-montanus* occurs in mixed conifer where *Picea* occurs, often in combination with *Abies* and *Pinus*. *C. elegantio-occidentalis* is found at higher elevations in the Cascade Mountains in mixed conifer forests with *Abies, Pinus,* and *Picea, Abies, Pinus* and *Pseudotsuga,* or *Abies, Picea, Pinus, Pseudotsuga* and *Larix*; at mid-elevations with *Abies*, *Pseudotsuga Tsuga*; and in coastal populations with *Picea* and *Tsuga.* The color reactions of fresh specimens with KOH in *C. elegantio-montanus* and *C. elegantio-occidentalis* range from

pinkish to reddish in the flesh of the stipe base which is similar to European populations. Usually the pileus surface also gives a reddish reaction although it may not be distinctly red in some instances. One of the characters emphasized in *C. elegantior* var. *americanus* was the lack of a change in the color of the flesh in the stipe base from to "rhubarb color" after exposure [4], a characteristic of the European collections. Observations on a number of collections from different areas of western North America give mixed results for this characteristic. Sometimes a pinkish to reddish flush in the stipe base is readily seen while in other instances it is very slow to develop or does not develop at all (requires observation after cutting for 15 minutes or longer). Coloration of the basidiomata is similar across the western North America species, with the amount of yellow color in the veil, lamellae and on the pileus surface varying somewhat from collection to collection. Our observations indicate that coloration is not a useful feature in separating these species from one another.

Basidiospore size overlaps with the length and width measurements given for European populations, however, in general the basidiospores tend to be somewhat shorter and narrower in some western North American populations. For example, JFA 11693 had on average smaller spores than usual (11.5–) 12 – 13.5 (–14.5) x (6.5–) 7 – 7.5 (8.5) µm, as compared to other samples of *C. elegantio-occidentalis* (12–) 12.5 – 15.5 (–16.5 – 18) x (7–) 7.5 – 9 (10) µm. In Europe, based on differences of the shape of the stipe base and veil development Moser (1960) [1] recognized two varieties of *C. elegantior*, namely *C. elegantior* var. *eduliformis* and *C. elegantior* var. *volvatum*. We sequenced the collection IB20040204 identified as *C. elegantior* var. *eduliformis* by M. Moser that resulted in

identical ITS sequences with European populations of *C. elegantior*. In Europe, *C. elegantior* is rather widely distributed and occurs commonly on calcareous soil between the 400 to 1300 m associated with *Picea abies*.

Cortinarius napus **Fr. Epicr. Syst. Mycol. (Upsaliae): 263 (1838).**

Synonyms:

C. albobrunnoides var. *albobrunnoides* M. M. Moser & McKnight, Mycotaxon **55**:301–346 (1995).

C. albobrunnoides M. M. Moser & K. McKnight var. *violaceovelatus* M. M. Moser & J. Ammirati, Mycotaxon **58**: 390–391 (1996).

C. subpurpureophyllus A. H. Smith var. *sulphureovelatus* M. M. Moser, Mycotaxon **74**(1): 25 (2000).

Pileus 35 – 170 mm diam., convex or more expanded, margin at first involute, radially innately fibrillose, not hygrophanous, viscid, sometimes with patches of veil tissue along margin, color of margin pale ochraceous to pale brown or orange brown, at centre brown, becoming dark red brown. *Lamellae* adnexed, crowded, edges serrated, color young greyish white, sometimes lilac at first, in age greyish brown to dark medium brown. *Stipe* 32 – 150 mm long, 8 – 35 mm thick above, with a rounded or only slightly marginate bulb (bulb 14 – 50 mm thick), color whitish to ochre or brownish, sometimes pale lilac to bright lilac at first, bulb margin white to lilac, then brownish ochraceous, sometimes with violet veil on rim. *Basal mycelia* of bulb white, pale yellow, light yellow or lemon yellow

to greenish yellow. *Context* white, slightly yellow in the stipe base, sometimes lilac in cortex of stipe down to bulb. *Odor* none or slightly pungent. *Taste* mild, slightly astringent or slightly raphanoid.

Basidiospores 10.5 – 14 x 6.5 – 8 µm, Q = 1.50 – 1.87, mean = 1.64 and S = ± 0.10, amygdaliform to citriniform, verrucose. *Basidia* 30 – 40 x 8 – 12 µm, 4-spored, clavate, colorless. *Lamella trama* regular composed of hyphae cylindrical, 3 – 24 µm wide, colorless. *Pileipellis* simplex. *Gelatinous layer of pileus* composed of hyphae 3 – 10 µm wide, colorless to yellowish or brownish, sometimes encrusted. *Hyphae of epicutis* 4 – 16 µm wide, cylindrical, hyaline to brown, faintly to distinctly spirally encrusted, sometimes with epiparietal incrustations. *Hyphae of pileus trama*, cylindrical, 4 – 26 (–34.5) µm, hyaline to pale yellow or rarely brownish. *Veil hyphae* 2.5 – 6.5 µm wide, cylindrical, hyaline to slightly yellowish, a few slightly encrusted. Oleiferous hyphae sometimes present, yellowish to greyish. *Clamp connections* present.

Macrochemical reaction: Fresh specimens: Pileus surface grey brown to brownish or dark brown, pileus context negative to pale brownish. Dried specimens: Pileus surface red brown, greyish in pale colored specimens, and basal mycelia pink.

Specimens examined: Europe. **Germany**. Blasien-Dachsberg (47.729722, 8.098889), *Picea abies*, November 01, 2004, TUB 012717 (EU057067); St. Blasien-Liberg, *Picea abies*, November 04, 2005 TUB 019281 (GU363479); Löffingen (47.883889, 8.343611), *Abies alba*, *Picea abies*, October 17, 2005 TUB 019282 (GU363480). North America. **USA.** Colorado. Boulder County. Mountain Research Station, *Abies lasiocarpa, Picea engelmannii*, *Pinus contorta,* August 18, 1997, JFA 12426 (GU363481); Mountain Research Station near Nederland (39.961986, -105.510604), *Abies lasiocarpa*, *Picea*

engelmannii, August 18, 1997, IB19970194 (GU363488). Oregon. Lincoln County. Fogarty Creek Campground/State Park, Lincoln Beach (44.8425, -124.04444), gregarious, *Pseudotsuga menziesii*, *Thuja occidentalis*, *Tsuga heterophylla,* November 10, 1995, JFA 11723 (= IB19950515) (EU057016). Washington. Chelan County. Lake Ann trailhead, Rainy Pass, parking area (48.5240263, -120.6542698), caespitose to gregarious, *Abies lasiocarpa, Picea engelmannii*, *Pseudotsuga menziesii, Tsuga mertensiana,* September 12, 1989, JFA 10070 (EU057015). Wyoming. Fremont County. Shoshone National Forest, Union Pass, Trail to Union Peak, *Picea engelmannii*, August 21, 1989, IB19890298 (GU363483); 5 miles north of Union Pass (near road), Shoshone National Forest, *Picea engelmannii*, *Pinus contorta*, August 13, 1991, IB19910237 (GU363484); Union Pass (43.665, -110.066667), Shoshone National Forest, *Picea engelmannii*, *Pinus contorta*, August 15, 1991, IB19910270 (GU363486). Teton County. Flagstaff Road, 5.8 miles from HW, *Picea engelmannii, Pinus contorta*, August 6, 1997, JFA 12401; Flagstaff Creek near dam, Teton National Forest, *Abies lasiocarpa*, *Picea engelmannii*, August 10, 1997, IB19970162 (GU363487); Two Ocean Mountain (43.7411, -110.0863), Shoshone National Forest, *Picea engelmannii*, August 31, 1997, IB19970303 (GU363489) and IB1997303b (GU363491); Flagstaff Road (43.473998, -110.763878, for Togwotee Mountain Lodge) ca. 1 mile east on road, Teton National Forest, *Picea engelmannii*, August 09,1987, IB19870186 (GU363490).

Additional specimens examined: Europe. **Sweden.** Mockfjärd, Dalarna (60.5, 14.966667), *Picea abies*, October 07, 2004, S: F44393 (EU057069). North America. **USA.** Wyoming. Teton National Forest, Union Pass, Lake of the Woods, Windriver Mountains, 2800m, *Picea engelmannii*, August 22, 1987, IB19870275 (GU363482) and

August 15, 1991, IB19910261 (GU363485). Trail to Turbid Lake, (1st mile), Yellowstone National Park, *Pinus contorta*, August 16, 1989, IB19890242 (GU363492). Washington. Rainy Pass, Chelan City, *Abies lasiocarpa*, *Picea engelmannii*, September 12, 1989, IB19890479 (GU363493).

Comments: There are some morphological differences between disjunct populations of *Cortinarius napus*, especially concerning the coloration of the lamellae and veil. In European populations the lamellae of *C. napus* have been described as greyish coloured (e.g. [1]), but according to our field observations some populations can also have lilac lamellae. However, these characteristics need further documentation through the range of the species in Europe. In the western North American counterpart, populations of *C. napus* exhibit some variation of the violet pigmentation of the basidiomata from population to population; it may or may not develop on the stipe and lamellae. Earlier the pink KOH reaction of the basal mycelium of the stipe was overlooked [1], perhaps because of the lack of a well-developed basal mycelium in some European specimens as compared with their disjunct North American counterparts [4,6,7,8]. In western North American the basal mycelium can be strongly yellowish to greenish yellow. Commonly, European populations of *C. napus* occur as a few individuals and the populations are somewhat infrequent. Therefore this species has a rather fragmentary distribution in Europe being known with certainty from some sites in Norway, Sweden, France and Germany [1,9]. Our three collections from Germany were from calcareous soil with *Picea*, in one site with *Abies*. Western North American populations of *C. napus* in the Rocky Mountains occur in conifer forests with *Picea*, where *Abies* and *Pinus* may be present. In the Cascade Mountains of Washington it occurs in mixed conifer forests of

Abies, Picea, Pseudotsuga and *Tsuga*, while in coastal Oregon and northern California the species is known from low elevations with *Pseudotsuga, Picea,* or *Pseudotsuga* and *Tsuga*.

The phylogenetic analysis supports the members of this complex as an isolated line of speciation within the section *Calochroi* (data not shown).

Methology: Macroscopical descriptions are based on fresh specimens, whereas microscopical examinations are from dried specimens mounted in 3% KOH and studied using bright field microscopy. Macrochemical tests were done on both fresh and dried specimens using 40% KOH; tests using other concentrations of KOH are given in the text. Color notations are from Ridgway, *Color Standards and Nomenclature*, capitalized, Ochraceous Tawny, Munsell, Mu7.5YR6/7, Methuen, Me7A8, and Cailleux, K53. Collections of Joe Ammirati (JFA) are deposited in the Burke Museum, Herbarium, University of Washington (WTU) unless otherwise indicated in the cited materials.

References

1 Moser M: ***Die Gattung Phlegmacium (Schleimköpfe)***. Julius Klinkhardt, Bad Heilbrunn, Germany; 1960.

2 Moser M, Ammirati JF: **Studies on North American Cortinarii IV. New and interesting *Cortinarius* species (subgenus *Phlegmacium*) from oak forests in Northern California.** *Sydowia* 1997, **49**:25–48.

3 Frøslev TG, Jeppesen TS, Laessøe T, Kjøller R: **Molecular phylogenetics and delimitation of species in *Cortinarius* section *Calochroi* (Basidiomycota, Agaricales) in Europe.** *Molecular Phylogenetics and Evolution* 2007, **44:**217–227.

4 Moser M, McKnight KH, Ammirati JF: **Studies on North American Cortinarii I. New and interesting taxa from the Greater Yellowstone area.** *Mycotaxon* 1995, **55**:301–346.

5 Brandrud TE, Lindström H, Marklund H, Melot J, Muskos S: ***Cortinarius, Flora Photographica.*** *(German Version). Cortinarius HB, Matfors 1990-Vols 1 (1990), 2 (1992), 3 (1995) & 4 (1998)*. 1998.

6 Smith AH: **Studies in the genus *Cortinarius* I.** *Contributions from the University of Michigan Herbarium* 1939, **2**:1–42.

7 Moser M, Ammirati JF: **Studies in North American Cortinarii VI. New and interesting taxa in subgenus *Phlegmacium* from the pacific states of North America.** *Mycotaxon* 2000, **74**:1–36.

8 Moser MM, Ammirati JF: **Studies in North American Cortinarii II. Interesting and new species collected in the North Cascade Mountains, Washington.** *Mycotaxon* 1996, **58**:387–412.

9 Soop K: ***Cortinarius in Sweden.*** Scientrix, Stockholm, Sweden; 2002.
